# Microbial-Related Metabolites May Be Involved in Eight Major Biological Processes and Represent Potential Diagnostic Markers in Gastric Cancer

**DOI:** 10.3390/cancers15215271

**Published:** 2023-11-03

**Authors:** Siru Nie, Ang Wang, Xiaohui Chen, Yuehua Gong, Yuan Yuan

**Affiliations:** 1 Tumor Etiology and Screening Department of Cancer Institute and General Surgery, The First Hospital of China Medical University, Shenyang 110001, China; niesiru@126.com (S.N.); quantax@outlook.com (A.W.); chenxiaohui516@163.com (X.C.); 2 Key Laboratory of Cancer Etiology and Prevention in Liaoning Education Department, The First Hospital of China Medical University, Shenyang 110001, China; 3 Key Laboratory of GI Cancer Etiology and Prevention in Liaoning Province, The First Hospital of China Medical University, Shenyang 110001, China

**Keywords:** gastric cancer, 16S rRNA, metabolites, microbes, biomarker

## Abstract

**Simple Summary:**

This article integrated analyses of the microbial and metabolic characteristics of gastric cancer (GC), as well as their coexistence relationship, and determined the microbial-related metabolites diagnostic markers. Our study provides a direction for finding microbial-related metabolic diagnostic markers of GC and may provide a basis for further understanding and exploring the coexistence and interaction mechanisms of microbe and metabolites in GC.

**Abstract:**

Metabolites associated with microbes regulate human immunity, inhibit bacterial colonization, and promote pathogenicity. Integrating microbe and metabolome research in GC provides a direction for understanding the microbe-associated pathophysiological process of metabolic changes and disease occurrence. The present study included 30 GC patients with 30 cancerous tissues and paired non-cancerous tissues (NCs) as controls. LC-MS/MS metabolomics and 16S rRNA sequencing were performed to obtain the metabolic and microbial characteristics. Integrated analysis of the microbes and metabolomes was conducted to explore the coexistence relationship between the microbial and metabolic characteristics of GC and to identify microbial-related metabolite diagnostic markers. The metabolic analysis showed that the overall metabolite distribution differed between the GC tissues and the NC tissues: 25 metabolites were enriched in the NC tissues and 42 metabolites were enriched in the GC tissues. The α and β microbial diversities were higher in the GC tissues than in the NC tissues, with 11 differential phyla and 52 differential genera. In the correlation and coexistence integrated analysis, 66 differential metabolites were correlated and coexisted, with specific differential microbes. The microbes in the GC tissue likely regulated eight metabolic pathways. In the efficacy evaluation of the microbial-related differential metabolites in the diagnosis of GC, 12 differential metabolites (area under the curve [AUC] >0.9) exerted relatively high diagnostic efficiency, and the combined diagnostic efficacy of 5 to 6 microbial-related differential metabolites was higher than the diagnostic efficacy of a single feature. Therefore, microbial diversity and metabolite distribution differed between the GC tissues and the NC tissues. Microbial-related metabolites may be involved in eight major metabolism-based biological processes in GC and represent potential diagnostic markers.

## 1. Introduction

The microbiota serves an important role in the mucosa of the gastrointestinal tract [1,2,3]. Several studies have demonstrated that microbes cause DNA damage, promote tumorigenesis, and create a tumor-promoting environment by affecting the immune system [4,5]. In recent years, an increasing number of studies have focused on microbes in gastric cancer (GC) and identified significant differences between GC tissues and those of other gastric diseases [6,7,8,9]. Our previous studies showed that the microbiota in GC tissues and adjacent non-cancerous (NC) tissues differed in distribution, microbe diversity, and predicted metabolic function. The more abundant microbiota in GC tissues may play important roles in human biosynthesis, molecular decomposition, immune function, and disease occurrence [10]. In stomach diseases, it is widely accepted that *Helicobacter pylori* (*H. pylori*) plays an important role. However, only 3% of *H. pylori*-infected individuals eventually develop GC [11]. Previous studies also pointed out the decreasing colonization of *H. pylori* in the development of GC and the other bacterial contributions to GC progression [12,13]. Therefore, microbiota—other than only *H. pylori*—may play a more important role in GC.

Compared to other omics, changes in metabolomics could directly reflect the physiological state of humans. With the development of omics technology in recent years, non-targeted metabolomics has detected more metabolites, reflecting more relevant information. Metabolites associated with microbes regulate human immunity [14], inhibit bacterial colonization [15], and promote pathogenicity [16], such as colorectal cancer tumorigenesis [17,18]. Therefore, the metabolic function of the microbiota may be more important than its taxonomic composition [19]. Previous integrated analysis showed that microbes act as biochemical converters to disrupt the metabolic balance in colorectal cancer tissues [20]. Thus, the resulting metabolite changes establish an important connection between cancer and intestinal microbes [17,18]. The metabolomic-integrated intestinal microbes may also be immunotherapy biomarkers for colorectal cancer [19,21]. The microbiota changes in sterile mice, caused by injecting one or two bacteria, can affect the metabolite composition in various organs [22,23]. In irritable bowel syndrome (IBS) and inflammatory bowel disease (IBD), the integrated analysis of microbes and metabolites determined the main microbial metabolic pathways involved in the occurrence of these diseases [24,25]. In another study, the gut microbiota changes in HIV-infected patients were closely related to plasma metabolism disorders [26].

Integrating microbe and metabolome research provides a direction for understanding microbe-associated pathophysiological processes in metabolic change and disease occurrence [15,27,28]. Currently, GC studies mostly focus on one kind of omics, either mining the changes in the microbial community and abundance or exploring the differences in metabolites and metabolic pathways. Metabolites in serum or tissues, without the involvement of microbes, might serve as biomarkers for identifying GC [29,30]. However, there are no reports on the correlation between these two omics in GC. 

The present study aimed to determine the microbial and metabolic characteristics that mainly affected GC, using 16S rRNA and non-targeted metabolomics. Under the differential analysis of microbes and metabolites, a neural network algorithm was utilized to predict the response intensity of metabolites, given a single-input microbial sequence, in order to predict the coexistence relationship between microbes and metabolites [31]. This analysis provides directions for finding GC-related microbial and metabolic diagnostic markers and a basis for further understanding the relationship between microbial and metabolic functions in GC tissues, as well as a basis for exploring their coexistence and interaction mechanisms.

## 2. Materials and Methods

### 2.1. Sample Collection

This study included 30 GC patients who underwent subtotal gastrectomy from June 2012 to June 2014 in the First Affiliated Hospital of China Medical University. The inclusion and exclusion criteria were consistent with our previous research criteria [10]: 30 GC tissues and paired NC tissues were obtained from 30 GC patients who underwent subtotal gastrectomy; the patients had clear postoperative pathological diagnoses as advanced and low-median differentiated gastric adenocarcinoma, via both hematoxylin and eosin (H&E) staining and immunohistochemistry; patients who had received medical treatment (including probiotics, proton pump inhibitors, antibiotics, and H2 receptor antagonists) within 1 month were excluded; patients who had received chemotherapy or radiotherapy prior to the surgery were excluded. Fresh gastric mucosal tissues collected from the lesion and normal distal sites were immediately frozen after the operation and stored at −80 °C. The study was approved by the Ethics Committee of the First Affiliated Hospital of China Medical University, and samples were collected with informed consent.

### 2.2. Process and Analysis of Metabolomics in GC and NC Tissues

#### 2.2.1. Metabolite Extraction from Tissues

Tissues (100 mg) were pulverized with liquid nitrogen, then resuspended in 500 μL 80% prechilled methanol containing 0.1% formic acid. After vortexing thoroughly, the samples were incubated on ice for 5 min and centrifuged at 15,000× *g* for 10 min at 4 °C. The supernatant was diluted with LC-MS-grade water to a final concentration of 53% methanol, transferred to a fresh Eppendorf tube, and centrifuged at 15,000× *g* for 10 min at 4 °C. The supernatant was analyzed by LC-MS/MS [32]. A mixed sample of each experimental sample with an equal volume was used as a quality control (QC) sample. A 53% methanol aqueous solution containing 0.1% formic acid was used as a blank sample.

#### 2.2.2. Untargeted Metabolome Analysis by LC-MS/MS

Metabolome analysis was performed by Novogene Co., Ltd. (Beijing, China) with a Vanquish ultra-high performance liquid chromatography-mass spectrometry (UHPLC-MS/MS) system (Thermo Fisher, Karlsruhe, Germany) coupled to an Orbitrap Q Exactive™ HF mass spectrometer (Thermo Fisher, Germany). The separation was performed on a C18 Hypersil Gold column (Thermo Fisher, Germany), using a 17-min linear gradient at an 0.2 mL/min flow rate. The solvent gradient of the eluents for the positive polarity mode (eluent A: 0.1% formic acid in water; eluent B: methanol) and the negative polarity mode (eluent A: 5 mM ammonium acetate, pH 9.0; eluent B: methanol) was set as follows: t = 0 min, 2% B; t = 1.5 min, 2% B; t = 12 min, 2–100% B; t = 14 min, 2–100% B; t = 14.1 min, 100–2% B; t = 17 min, 2% B. The Q Exactive™ HF mass spectrometer was operated with a spray voltage of 3.2 kV, a capillary temperature of 320 °C, a sheath gas flow rate of 40 arbitrary units, and an aux gas flow rate of 10 arbitrary units. The scanning range for ion separation was 70–1050 *m*/*z*, at a resolution of 60,000. An automatic gain control (AGC) of 3 × 10^6^ with a maximum ion injection time of 100 ms was set for the Orbitrap parameters.

#### 2.2.3. Data Processing and Metabolite Identification for the Metabolome Analysis

The raw data generated by UHPLC-MS/MS were processed by Compound Discoverer 3.1 (CD3.1, Thermo Fisher). Peak alignment, peak picking, and quantitation for each metabolite were performed with the following parameters: retention time tolerance, 0.2 min; actual mass tolerance, 5 ppm; signal intensity tolerance, 30%; signal/noise ratio, 3; minimum intensity, 100,000 ppm. Peak intensities were normalized to the total spectral intensity for predicting the molecular formula, based on additive ions, molecular ion peaks, and fragment ions. The peaks were matched with the mzCloud (https://www.mzcloud.org/, accessed on 28 April 2020), mzVault and MassList databases to obtain accurate qualitative and relative quantitative metabolite results. Statistical analyses were performed using the statistical software R (R version R-3.4.3), Python (Python 2.7.6 version), and CentOS (CentOS release 6.6).

#### 2.2.4. Metabolite Annotation, Screening, and Differential Metabolite Analysis

Metabolites were annotated using the HMDB database (https://hmdb.ca/metabolites, accessed on 15 May 2020). Principal components analysis (PCA) and partial least squares discriminant analysis (PLS-DA) were performed to obtain the overall distribution trend of the tissues and differential metabolites using metaX, a flexible and comprehensive software for processing metabolomics data. The *t*-test and PLS-DA were used to screen the differential metabolites coordinately with the filtered conditions of the variable importance for the projection (VIP) > 1.0, fold change (FP) 2.0 or <0.5, and *p* < 0.05.

### 2.3. Sequencing and Analysis of the Microbiome in GC and NC Tissues

#### 2.3.1. 16S rRNA Sequencing and Data Processing

Genomic DNA was extracted from the GC tissues and the NC tissues for the quality inspection, amplification, and sequencing of the V4–V5 region of the 16S rRNA gene. After sequencing, the raw data were split, intercepted, spliced, and filtered to obtain relatively valid data. Genomic DNA extraction and gene sequencing were performed, as previously described [10]. Subsequently, amplicon sequence variants (ASVs) data were obtained by the DADA2 denoising method, using the QIIME 2 2020.2 platform. The microbes were annotated by comparing the ASVs data to the Silva database, with 99% similarity [33,34].

#### 2.3.2. Analysis of Microbial Diversity Differences and Differential Bacteria through 16S rRNA Sequencing

The analysis of microbial diversity differences contained α and β diversity. The α-diversity index (Richness, Chao1, phylogenetic diversity [PD] whole tree indices, Shannon, Simpson, and Pielou) results were calculated based on the ASVs data, using the R language vegan package. The β-diversity metrics (weighted UniFrac, unweighted UniFrac, Jaccard distance, and Bray–Curtis dissimilarity) were calculated using the QIIME 2 platform q2-diversity plugin. The differences between the two groups were compared using permutational multivariate analysis of variance (PERMANOVA) [35,36]. The *t*-test and the linear discriminant analysis effect size (LEfSe) tool were used to screen the differential microbes between the two groups, with the following filters: log2FC > 1, LDA (linear discriminant analysis) > 2, and *p* < 0.05.

### 2.4. Microbe-Metabolite Correlation Analysis in GC and NC Tissues

#### 2.4.1. The Overall Correlation Analysis of the Microbes and Metabolites

The correlation analysis of the microbes and metabolites was performed using the M2IA website (http://m2ia.met-bioinformatics.cn/, accessed on 7 July 2020) [37], including the Procrustes overall similarity analysis and the O2PLS model of matrix correlation analysis.

#### 2.4.2. Coexistence Analysis of Differential Microbes and Metabolites

The coexistence relationship and the probability of the differential microbes and metabolites between the GC tissues and the NC tissues were analyzed, using the microbe-metabolite vector (mmvec) neural network algorithm [31]. The microbes that contributed more to the richness of the metabolites were obtained by sorting, clustering, and visualizing the microbe–metabolite interactions, using dimension reduction analysis.

#### 2.4.3. Functional Enrichment Analysis of the Microbial-Related Metabolites

We used the Greengenes13.0 database to re-annotate the ASVs data to obtain microbe abundance data for the microbial-related metabolic function prediction of the GC tissues and the NC tissues. The metabolic prediction of the microbial community was performed using PICRUSt2 software (v2.3.0-b) and the Kyoto Encyclopedia of Genes and Genomes (KEGG) database, and included the nearest sequenced taxon index accuracy test [38], enrichment analysis of the KEGG metabolic pathways, and LefSe analysis of microbial metabolic function differences. The metabolic function analysis of the differential metabolites between the GC tissues and the NC tissues was performed using MetaboAnalyst 5.0 (https://www.metaboanalyst.ca/, accessed on 14 January 2021) through the KEGG database. A metabolic pathway had significant enrichment when *p* < 0.05.

### 2.5. Diagnostic Efficacy Analysis of Differential Microbial-Related Metabolites between GC and NC Tissues

Based on the coexistence analysis between the microbes and the metabolites, the receiver operating curve (ROC) was constructed to distinguish the differences in the characteristics between the differential microbial-related metabolites between the GC tissues and the NC tissues by the R language pROC package. Metabolites with AUC > 0.9 were used as potential metabolite features for the random forest analysis. The random forest model was constructed and trained to obtain their importance ranking for searching for the most combination models that could distinguish the GC tissues from the NC tissues.

### 2.6. Statistical Analysis

For the microbial analysis, α-diversity differences were calculated by the Mann–Whitney U test, using SPSS 25.0 software (SPSS Inc., Chicago, IL, USA). Differences in β-diversity were calculated using PERMANOVA. In the analysis of differential bacteria and predicting metabolic functions, the screening conditions satisfied log2FC > 1 and *p* < 0.05 for the *t*-test and LDA > 2 and *p* < 0.05 for LEfSe. For the metabolite-associated analysis, the screening conditions for the differential metabolites satisfied log2FC > 1 and *p* < 0.05 for the univariant analysis and VIP > 1.0 and *p* < 0.05 for PLS-DA. The volcano map was drawn in R language. Pearson correlation analysis was performed to predict the relationship between differential metabolites. The metabolic pathway network analysis for the differential metabolites was performed using Cytoscape. The ROC and the random model were constructed using the R language. Graphs were drawn using Prism GraphPad 8.4.1 and R language. *p* < 0.05 was considered statistically significant.

## 3. Results

### 3.1. General Sample Information

Thirty patients (eight women and 22 men, aged 40 to 83) with a pathological diagnosis of GC were included in this study. The average age of these patients was 62 years (median age 63 years). Fifteen patients used to smoke and 13 patients used to drink. All enrolled patients provided signed informed consent (Supplementary Table S1).

### 3.2. The Metabolic Characteristics in the GC Tissues and the NC Tissues

#### 3.2.1. The Overall Distribution of Metabolites in the GC Tissues and the NC Tissues

**Figure 1:**
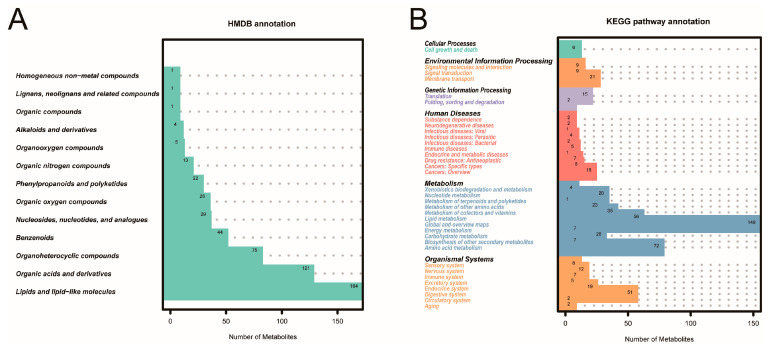
The annotation of metabolites from 30 GC patients. (**A**) The annotation in the HMDB database. (**B**) The annotation in the KEGG database.

Based on non-targeted metabolomics by LC-MS/MS, we compared the molecular feature peaks with the database to obtain metabolites, and we retained the metabolites with a coefficient of variation of less than 30% in the QC samples. Then, we qualified and quantified 496 negative and 863 positive ion metabolites in the GC tissues and the NC tissues. Subsequently, the metabolites were annotated using the HMDB and KEGG databases. In the HMDB database, 508 metabolites in 13 superclasses were annotated, including organic compounds and lipid molecules (e.g., lipids, organic acids, and organic heterocyclic compounds) (Figure 1A). In the KEGG database, these metabolites were mainly involved in the overall metabolic process, including amino acid, lipid, vitamin, and nucleotide metabolism (Figure 1B).

#### 3.2.2. Differential Metabolites between the GC Tissues and the NC Tissues

**Figure 2:**
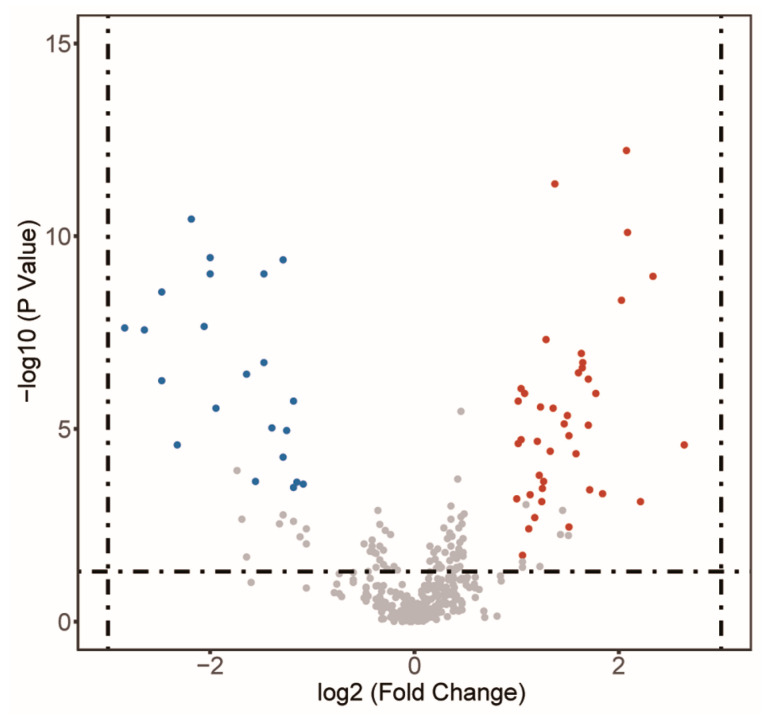
Differential metabolites between GC tissues and NC tissues, by PLS-DA. Forty-two red points demonstrated metabolites enriched in GC tissues; 25 blue points demonstrated metabolites enriched in NC tissues.

**Figure 3:**
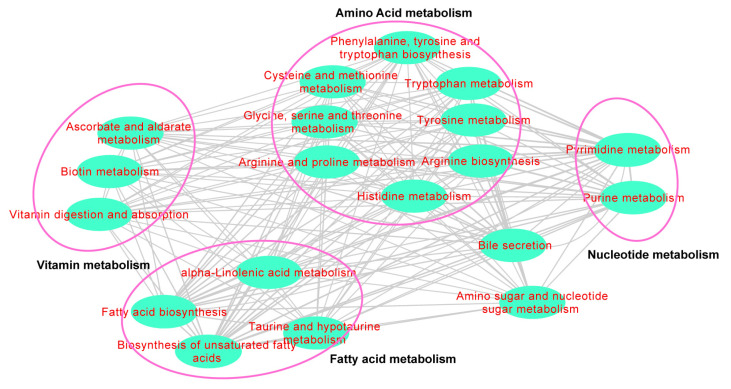
Metabolite processes of 67 cancer differential metabolites. Each line demonstrated the relationship between metabolites. The metabolite processes were drawn and enriched by Cytoscape software (v3.8.0).

Based on the metabolites and their categories annotated in the HMDB database, we compared the overall distribution and differences in metabolites between the GC tissues and the NC tissues. PCA analysis revealed that the overall metabolite distribution differed between the two tissue types (Supplementary Figure S1). Based on the rank sum test, there were 230 differential metabolites between the two groups (*p* < 0.05), including lipids, alcohols, phenols, alkylamines, and organic acids (Table S2). PLS-DA analysis was then performed to process data through dimensionality reduction, regression analysis, and model creation to identify the differential metabolites with significant differences. The results indicated that the model was not overfitting (Figure S1). After setting a strict screening threshold (VIP > 1.0, FC > 2.0 or FC < 0.5, *p* < 0.05), 25 metabolites were enriched in the NC tissues and 42 metabolites were enriched in the GC tissues (Figure 2, Table 1). These sixty-seven metabolites were correlated with GC; these metabolites were involved in amino acid, fatty acid, nucleotide, and vitamin metabolism (Figure 3).

### 3.3. The Microbial Characteristics in GC Tissues and NC Tissues

#### 3.3.1. The Overall Microbe Composition and Microbial Diversity in GC Tissues and NC Tissues

The overall microbe composition was obtained using the Qiime2 microbiome bioinformatics platform, based on our previous 16S rRNA sequencing results. In GC tissues and NC tissues, the enriched bacteria phyla included *Proteobacteria*, *Firmicutes*, *Bacteroidetes*, and *Epsilonbacteraeota* (Figure S2). We calculated the differences in α and β diversity of the microbes between the GC tissues and the NC tissues. Compared with the NC tissues, the α diversity indexes (richness, Chao1 index, faith-PD_whole_tree, Shannon, Simpson, and Pielou uniformity) in the GC tissues were higher, indicating a higher richness, diversity, and genetic diversity (*p* < 0.05, Figure S3). After the visualization by Principal coordinates analysis (PcoA), the β diversity distance metrics for the unweighted UniFrac (*p* = 0.001) and the Jaccard_distance (*p* = 0.001) showed statistical differences, indicating a difference in microbial structure diversity between the GC tissues and the NC tissues (Figure S4).

#### 3.3.2. Differential Microbes in GC Tissues and NC Tissues

**Figure 4:**
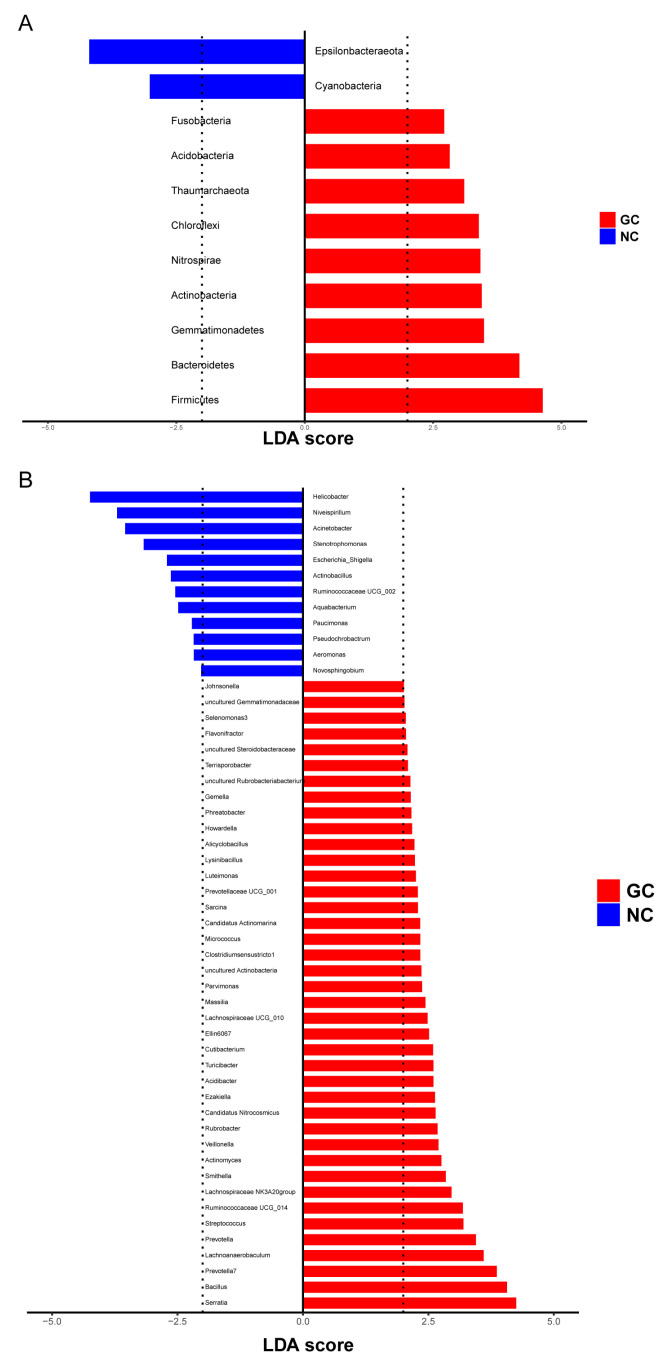
Differential microbe in phyla and genera between GC tissues and NC tissues by LefSe. (**A**) Differential phyla between GC tissues and NC tissues. (**B**) Differential genus between GC tissues and NC tissues.

Based on the microbial diversity results, we further analyzed the differential microbes by phyla and genera, using the *t*-test and LEfSe. Eight bacterial phyla and 29 bacterial genera were enriched in the GC tissues, compared to the NC tissues (Table S3). Using LEfSe, we screened 11 differential phyla and 52 differential genera for the subsequent relevance analysis with the metabolites (Figure 4).

### 3.4. Correlation between Metabolites and Microbes in GC Tissues and NC Tissues

#### 3.4.1. The Overall Correlation between Metabolites and Microbes

**Figure 5:**
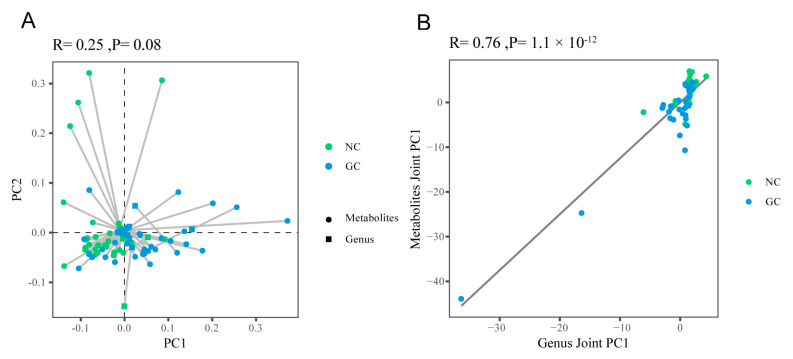
The overall correlation between metabolites and microbes. (**A**) Procrustes analysis. (**B**) O2PLS model.

To explore the relationship between metabolomics and the microbiome in GC, Procrustes analysis was performed to analyze the overall correlation between these two datasets (1659 metabolites and all genera by 16S rRNA sequencing), showing an overall similarity with no statistical significance (Figure 5A). After two-way modeling of the two omics matrices by the O2PLS model, we found that the metabolomics data matrix was highly correlated with the microbiome (r = 0.76, *p* < 0.05, Figure 5B), which provided a foundation for further association analysis.

#### 3.4.2. The Coexistence of Metabolites and Microbes in GC Tissues and NC Tissues

**Figure 6:**
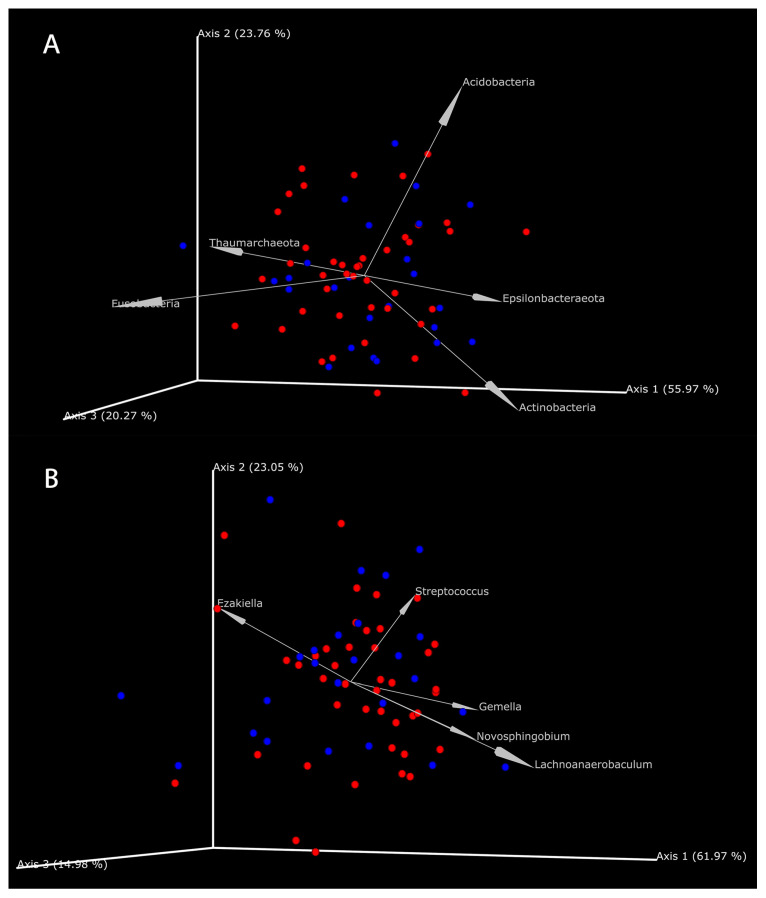
The coexistence relationship of differential metabolites with differential microbes. (**A**) The coexistence relationship of differential metabolites with differential phyla. (**B**) The coexistence relationship of differential metabolites with differential genus. Each figure showed the top 5 differential microbes. Red points represent the enriched metabolites in GC tissues; blue points represent the enriched metabolites in NC tissues. The smaller the angle between points and arrows, the greater the possible coexistence relationship between microbes and metabolites.

**Figure 7:**
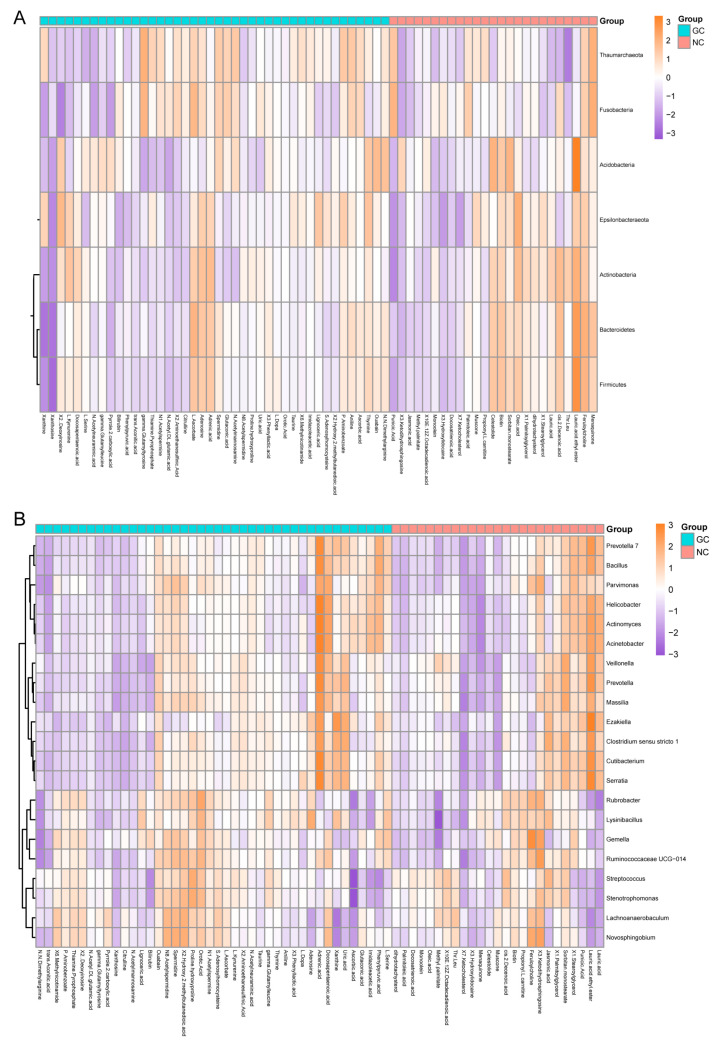
The coexistence probability of each differential metabolite and microbe. (**A**) The coexistence probability of differential metabolite and differential phyla. (**B**) The coexistence probability of differential metabolite and differential genus. The larger the positive log conditional probabilities, the stronger the likelihood of co-occurrence between microbes and metabolites, while the low and negative values indicated no relationship.

To further explore the coexistence relationships between the metabolites and microbes and to find the microbial-related metabolites in the GC tissues and the NC tissues, mmvec [31] analysis evaluated the conditional coexistence probability of differential metabolites with specific differential microbes, using a neural network algorithm for discovering the interaction distribution between differential metabolites and microbes. Differential microbes (11 differential phyla and 52 differential genera) were obtained from the LEfSe results, and differential metabolites (67 differential metabolites) were obtained from the strict analysis of the PLS-DA and the rank sum test results. At the level of bacteria phyla, *Acidobacteria*, *Fusobacteria*, *Actinobacteria*, *Thaumarchaeota*, *Epsilonbacteraeota*, and seven other phyla existed simultaneously with each other and might coexist with 62 metabolites. At the level of the bacteria genera, 21 genera (e.g., *Actinomyces*, *Bacillus*, *Gemella*, *Prevotella*, and *Helicobacter*) might coexist with 63 differential metabolites. Of these differential metabolites, only xanthosine had no coexistence relationship with any genus or phyla (Table S4). The top five coexisting phyla and genera are shown in Figure 6 and Figure S5. The coexistence probability of each metabolite and microbe is presented in Figure 7. The greater the positive conditional probability value, the greater the probability of coexistence.

#### 3.4.3. Functional Enrichment of Microbial-Related Metabolites in GC Tissues and NC Tissues

**Figure 8:**
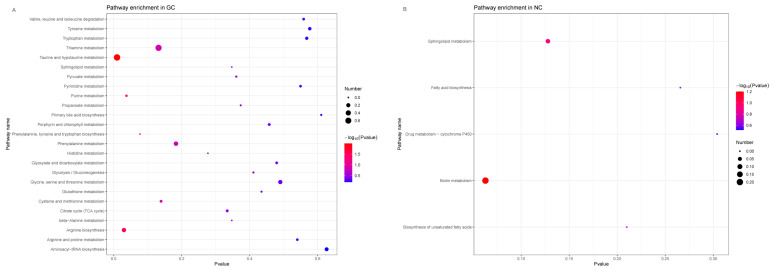
The KEGG pathway enrichment analysis of 66 microbial-related metabolites. (**A**) The KEGG pathway enrichment for microbial-related metabolites enriched in GC tissues. (**B**) The KEGG pathway enrichment for microbial-related metabolites enriched in NC tissues.

**Figure 9:**
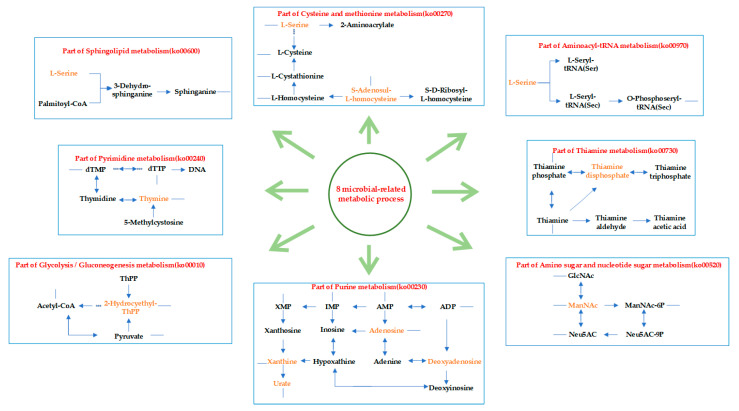
Eight major microbial-related metabolism-based biological partial processes in GC. Metabolites (yellow texts) were microbial-related metabolites hit on KEGG pathways by enrichment analysis, as well as ones mediated by differential microbiota.

The differentially enriched microbial-related metabolites identified in GC tissues and NC tissues participate in various metabolic pathways. We used MetaboAnalyst (https://www.metaboanalyst.ca/, accessed on 17 January 2021) to perform KEGG pathway enrichment for 66 microbial-related metabolites highly enriched in the GC tissues and the NC tissues. In the GC tissues, the main enriched pathways included taurine and hypotaurine metabolism, purine metabolism, and arginine biosynthesis. The main enriched pathways in the NC tissues included biotin metabolism, sphingolipid metabolism, and unsaturated fatty acid biosynthesis (Figure 8). As the microbial communities may cause pathway differences, we re-annotated the microbes with the Greengenes database to predict the microbial-related metabolic functions, using PICRUSt2 (Figure S6). By comparing the metabolic pathways predicted by the microbes and the pathways enriched by the microbial-related metabolites, eight metabolic pathways appear to be regulated by the microbes in GC (Table 2, Figure 9). 

### 3.5. Efficacy of Microbial-Related Differential Metabolites in the Diagnosis of GC

**Figure 10:**
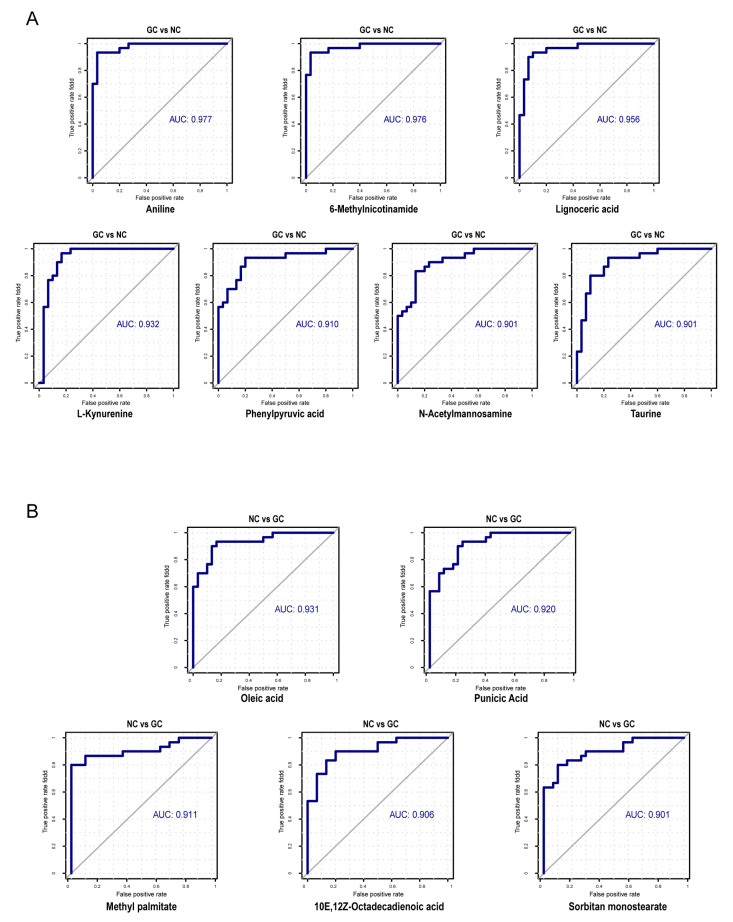
The ROC of microbial-related differential metabolites with high diagnostic efficiency (AUC > 0.9). (**A**) Seven microbial-related differential metabolites enriched in GC tissues for diagnosing GC. (**B**) Five microbial-related differential metabolites enriched in NC tissues for diagnosing NC.

**Figure 11:**
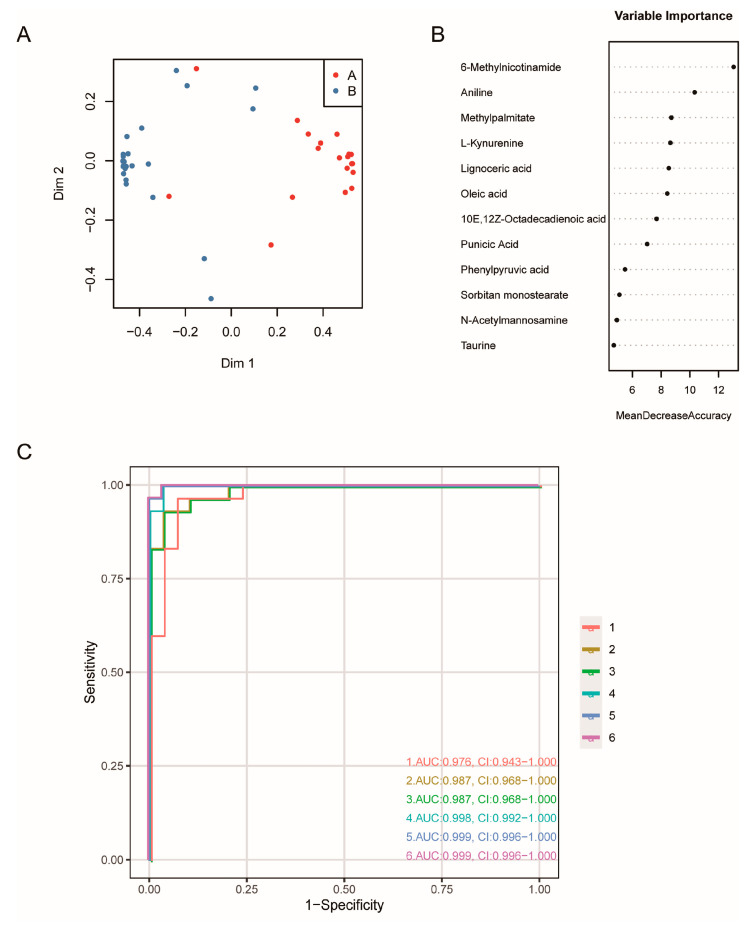
The random forest and ROC of microbial-related differential metabolites in the diagnosis of GC. (**A**) The random forest model. (**B**) The importance-ranking of microbial-related differential metabolites. (**C**) The combined diagnostic efficacy of microbial-related differential metabolites. For example, the number 6 represented the combined of top 6 microbial-related differential metabolites.

Based on the differences in metabolites between GC tissues and NC tissues, mentioned above, and the coexistence relationship between the metabolites and microbes, microbial-related metabolites may represent early-warning markers for GC. Therefore, we constructed an ROC model and calculated the AUC for microbial-related differential metabolites: 12 differential metabolites (AUC > 0.9) had relatively high diagnostic efficiency (Figure 10). Random forest analysis was used to model the microbial-related differential metabolites with high diagnostic efficiency and to analyze the best diagnostic efficacy of various metabolite combinations. The metabolite features with a mean decrease accuracy (MDA) greater than 8 were selected as candidate features for constructing the ROC and calculating the diagnostic efficacy with different combinations (Figure 11A,B). The results suggested that the combined diagnostic efficacy of 5 to 6 microbial-related differential metabolites was higher than the diagnostic efficacy of a single feature (AUC = 0.999) (Figure 11C).

## 4. Discussion

In the present study, the microbial and metabolic characteristics of 30 GC tissues and paired NC tissues were obtained through 16S rRNA gene and non-targeted metabolomics, with analysis of quality inspection, database comparison, and annotation. Through metabolomics analysis, we classified the overall characteristics of the metabolites in both tissue types, identified the differential metabolites, and determined metabolic network pathways for the two groups. Through Qiime2 microbiological analysis, we compared the overall microbial characteristics and microbial diversity of the GC tissues and the NC tissues and determined the differential microbes. Based on the characteristics of the microbiomes and metabolomes, we used mmvec to obtain the coexistence probability of differential microbes and metabolites and analyzed the metabolic functions of the microbial-related metabolites. The microbial-related metabolites were analyzed to determine their diagnostic efficacy for GC. These analyses showed that the GC tissues were more enriched in amino acids, organic acids, and some nucleotides, but less enriched in lipids, with higher microbial diversity than that of NC tissues. There were also differentially enriched microbes and metabolites in the GC tissues, with a high coexistence relationship. Moreover, microbial-related metabolites might participate in eight main metabolism-based biological processes that could be used as diagnostic markers for this disease. These results provide a basis for understanding the relationship and the interaction mechanism between microbes and metabolic functions.

### 4.1. Differential Metabolites between GC Tissues and NC Tissues Participated in the Process of Sugar, Amino Acid, Nucleotide, and Lipid Metabolism

As tissue/cell metabolism could affect biological organization, metabolites detected in tissues were closer to phenotypes (e.g., cell morphology, tumor type, tumor occurrence and development, genotype, and proteome) and could accurately and directly reflect the occurrence of life activities. Previous metabolomics studies have suggested that certain amino acids, organic acids, nucleotides, and lipids are increased in GC tissues [39,40,41]. The metabolites, which were qualified, quantitated, and annotated using non-targeted LC-MS/MS metabolomics, were more extensive than they were in previous studies, and contained more differential metabolites [39,40,41,42,43,44]. In the present study, the differential metabolites between GC tissues and NC tissues were mainly involved in sugar, amino acid, nucleotide, and lipid metabolism, providing directions for understanding the metabolic changes in GC.

Although the present study found no changes in metabolites directly involved in glycolysis, some changes were still related to sugar metabolism [39,40]. According to the Warburg Effect, even under conditions of sufficient oxygen, tumor cells prefer the energy supply from consuming glucose to generate ATP [45]. In addition to lactic acid, acetyl-CoA produced by glycolysis can participate in energy metabolism via the tricarboxylic acid (TCA) cycle. In our study, succinic semialdehyde (an intermediate metabolite for the production of succinate) and fumaric acid (a precursor to L-malate), which are involved in the TCA cycle, were both highly enriched in GC tissues; fumaric acid and malate were the most detectable metabolites in other studies [40,42,43,46,47,48].

A previous study suggested that an amino acid metabolism disorder might be closely related to the occurrence of GC [49], potentially caused by the upregulation of amino acid transporters during protein catabolism and the degradation of the extracellular matrix [47,50,51]. In the present study, due to the high enrichment of some protein hydrolysates (gamma-glutamylleucine and gamma-glutamyltyrosine) and protein modification metabolites (N,N-dimethylarginine and dipeptides [proline-hydroxyproline]) in GC tissues, we were more inclined to attribute the increase in amino acids to proteolysis. In addition, differential amino acids, such as leucine, asparagine, phenylalanine, tyrosine, proline, valine, threonine, glutamine, and proline, are also involved in the synthesis of the intermediate products of energy metabolism in the TCA cycle [44].

For nucleotide metabolism, purines and pyrimidines (e.g., xanthine, adenosine, 2′-deoxyinosine, thymine, and xanthosine), which are used to synthesize nucleotides [41,42], were highly enriched in GC tissues in the present study. Uric acid enrichment (nucleotide decomposition products) also provides evidence of active nucleotide metabolism [52,53]. Changes in amino acids (e.g., glutamine, glycine, and asparagine) cause an imbalance in the de novo synthesis of purine and pyrimidine, providing favorable growth conditions for cancer cells. The main manifestations of lipid metabolism in GC include the upregulation of mitochondrial fatty acid β-oxidation, the main energy source [44]. In the present study, a higher enrichment of acetylcarnitine in GC also suggested active fatty acid β-oxidation in this disease. In addition, the oxidative degradation of lipids in GC causes increased levels of 4-hydroxyphenylacetic acid [39]. Therefore, we speculated that GC cells had an increased demand for lipids, due to both active cell proliferation and active β-oxidative degradation of fatty acids, leading to decreased lipid levels and increased levels of oxidative decomposition products in the GC tissues. In summary, our analysis suggested that, compared with normal cells, GC cells had a better environment for proliferation, due to energy sources derived from glycolysis and amino acid, nucleoside, and lipid metabolism.

### 4.2. Metabolic Functions of Microbial-Related Metabolites

The changes in the metabolites and metabolic processes in GC tissues and NC tissues were affected by many factors, including the microbial community. Therefore, we performed microbial diversity analysis and differential microbial analysis to screen the differential microbes and coexistence relationship correlation analysis of the microbes and metabolites, to identify the microbial-related metabolites. The GC tissues showed a richer flora distribution, higher genetic diversity, and a more complex microbial structure than those of the NC tissues. Furthermore, changes in microbial communities in tissues depend on the microbial distribution at each level [6,7,10,54]. In the present study, bacteria at the phyla (*Thaumarchaeota*, *Acidobacteria*, *Actinobacteria*, *Bacteroidetes*, *Firmicutes*, and *Fusobacteria*) and genera (oral-related bacteria, such as *Fusobacterium*, *Streptococcus*, and *Prevotella*) levels were highly enriched in the GC tissues, whereas Helicobacter was enriched in the NC tissues. When the gastric mucosa has a different health status, the colonization and interaction of microbes and the relationship between the microbes and the host will change, resulting in differences in microbial diversity [8]. Therefore, the diversity changes observed between GC tissues and NC tissues in the present study could affect immune and metabolic processes [55,56]. Metabolic patterns in GC could be changed by the enrichment of some specific bacteria, resulting in tumor progression [57]. Meanwhile, microbiota could mediate drug resistance, resulting in metabolic disorders and influencing chemotherapy reactions [58,59]. In the analysis of the overall similarity between the microbes and metabolites, the metabolites were outside of the microbes, due to the high variability of the metabolites. In the correlation analysis of the two omics, the microbes were highly correlated with the metabolites in the GC tissues. Indeed, more than 90% of the differential metabolites could coexist with the specific differential bacteria, based on mmvec neural network algorithm analysis. However, only seven phyla and 21 genera coexisted with 66 differential metabolites. Therefore, the differential distribution of these seven phyla and 21 genera between GC tissues and NC tissues was most likely closely related to the differential metabolites.

We discovered eight microbial-related metabolic pathways through the intersection analysis of microbial metabolic function prediction and metabolite enrichment analysis, which mainly involved nucleotide and amino acid metabolic pathways. Based on the results of this analysis, we speculated that metabolic biological processes in GC were affected by those more enriched and major microbes by changing the metabolites of the amino acid and nucleotide metabolism pathways. Among the eight microbial-related differential metabolite enrichment pathways, those involved in the purine metabolism pathway belonged to the purine nucleoside-related metabolites (xanthine, adenosine, and deoxyinosine) and had different coexistence relationships with bacteria phyla. Previous studies suggested that gut microbes may be related to purine metabolism [60]. Bacterial genome-related studies also showed an asymmetry in the nucleotide composition of bacteria [61]. In the present study, *Firmicutes* and *Fusobacteria*, which have coexistence relationships with purine-related metabolites, showed obvious purine asymmetry (PAS) [62]. As a purine rescue enzyme, xanthine phosphoribosyl transferase (XPRT) in *Firmicutes* and *Bacteroidetes* could generate nucleotides from xanthine [63]. Therefore, we speculated that metabolites might act as an intermediate bridge between microbes and hosts to participate in the normal biological processes.

L-serine is a microbial-related metabolite involved in metabolism that may also play an important role in GC. L-serine is also the main source for the de novo synthesis of purine and deoxythymidine monophosphate during nucleotide metabolism and plays a central role in cell proliferation, indicating that L-serine in GC tissues may be involved in nucleotide metabolism to promote tumor cell proliferation. As a microbial-related metabolite, L-serine may also be a coexisting metabolite of *Acidobacteria*, *Actinobacteria*, and *Firmicutes* and participate in amino acid metabolism, such as the biosynthesis of aminoacyl tRNA and the cysteine and methionine metabolic pathways. In *Acidobacteria*, tRNA can function as regulatory non-coding RNA and amino acids, such as serine and cysteine, and can be effectively inserted into tRNA for subsequent biological activities. Twelve forms of tRNA were observed after combining with amino acids. Among these tRNAs, tRNA containing serine exists in *Actinobacteria* as tRNASec [64]. Therefore, the high enrichment of L-serine in the GC tissues may have a role in bacterial tRNA synthesis, and influence tissue metabolism by affecting the production of the corresponding amino acid. Therefore, if we start from the coexistence relationship between the microbes and metabolites, through the metabolic pathways involving microbial-related metabolites, we can uncover more information about microbial metabolism in GC tissues, predict the possible regulatory processes involving bacterial metabolic function, and perform a more in-depth molecular mechanism study combined with the current research on bacterial genomes.

### 4.3. The Diagnostic Potential of Microbial-Related Metabolites in GC

The present study evaluated the potential of microbial-related metabolites in diagnosing GC and found that 12 of these metabolites had an AUC over 0.9. The combination of the top six metabolites (6-methylnicotinamide, aniline, L-kynurenine, and lignoceric acid were highly enriched in GC; methyl palmitate and oleic acid were highly enriched in NC) in the random forest importance-ranking had higher diagnostic efficiency than that of each metabolite alone. Aniline, a metabolite coexisting with all seven bacteria phyla, was highly enriched in GC and had the potential to diagnose GC with an AUC as high as 0.97, which may be closely related to its carcinogenicity and mutagenicity [65,66]. L-kynurenine had a high coexistence probability with four bacteria phyla (*Actinobacteria*, *Bacteroidetes*, *Epsilonbacteraeota*, and *Firmicutes*), and previous studies demonstrated that it promotes tumor cell growth and migration [67]. In lung cancer, lignoceric acid can be used as a diagnostic marker [68,69]. In GC tissues, the low-enriched oleic acid may be associated with the decrease in lipids caused by tumor consumption or cachexia [39,40,46,70,71]. In mouse experiments, oleic acid levels were related to an imbalance in microbial distribution: with a high intake of oleic acid, the ratio of the bacteria phyla *Firmicutes* and *Bacteroidetes* decreased [72]. Therefore, oleic acid, a coexisting metabolite with many phyla (including *Firmicutes* and *Bacteroidetes*) in the present study, could be used as a diagnostic marker for GC. In general, microbial-related metabolites obtained in the present study provided excellent prediction efficiency. However, more validation about diagnostic efficiency comparison between microbial-related metabolites and other known markers for GC would be more convincing. 

In summary, based on the random forest prediction and the ROC curve, we speculate that highly differential microbial-related metabolites have the potential to diagnose GC; the combination of six microbial-related metabolites (6-methylnicotinamide, aniline, L-kynurenine, lignoceric acid, methyl palmitate, and oleic acid) had a high diagnostic efficiency for GC, close to 0.999. Our research provided new insights into microbial-related metabolites and their potential role in diagnosing GC. Changes in microbial-related metabolites and their clinical evaluation and application as diagnostic markers must be further explored.

## 5. Conclusions and Limitations

Microbial diversity and metabolite distribution were different between the GC tissues and the NC tissues. There were 66 microbial-related metabolites. Microbial-related metabolites may involve eight major metabolism-based biological processes in GC and represent potential diagnostic markers for this disease. However, the present study had some limitations. More in vitro validation about the relationship between these differential microorganisms and differential metabolites, more detection about alterations in microbial-related metabolic pathways, and more diagnostic efficiency comparison between microbial-related metabolites and other known markers for GC would be more convincing. In addition, we could not determine whether these metabolites are products of microbes or whether the metabolites change is influenced by microbes through current sequencing technology. Therefore, we predicted the coexistence probability to make predictions as reasonable as possible. We believe these challenges will be addressed with the development of new sequencing technology in the near future. 

## Figures and Tables

**Table 1:** Differential metabolites between GC tissues and NC tissues, by PLS-DA analysis.

	Name	FC	VIP
25 NC metabolites	Monoolein	0.04	1.76
	Palmitoleic acid	0.07	1.65
	1-Palmitoylglycerol	0.11	1.56
	Menaquinone	0.14	1.66
	dihydrotachysterol	0.16	1.66
	Sorbitan monostearate	0.18	1.71
	cis-2-Decenoic acid	0.18	1.71
	3-Hydroxylidocaine	0.2	1.12
	Oleic acid	0.22	1.85
	Lauric acid	0.24	1.67
	Punicic Acid	0.25	1.96
	Methyl palmitate	0.25	1.81
	Muscone	0.26	1.44
	7-Ketocholesterol	0.32	1.36
	Lauric acid ethyl ester	0.34	1.19
	3-Ketodihydrosphingosine	0.36	1.7
	Propionyl-L-carnitine	0.36	1.67
	1-Stearoylglycerol	0.38	1.26
	10E,12Z-Octadecadienoic acid	0.41	1.4
	Docosatrienoic acid	0.41	1.33
	Thr-Leu	0.42	1.75
	Jasmonic acid	0.44	1.34
	Feruloylcholine	0.44	1.23
	Biotin	0.45	1.14
	Celestolide	0.47	1.24
42 GC metabolites	2′-Deoxyinosine	2	1.1
	N-Acetylmannosamine	2.02	1.67
	N8-Acetylspermidine	2.02	1.55
	Docosapentaenoic acid	2.06	1.6
	N-Acetylneuraminic acid	2.06	1.38
	Thymine	2.08	1.09
	Adrenic acid	2.11	1.85
	2-Aminoethanesulfinic Acid	2.17	1.22
	Xanthine	2.19	1.26
	Orotic Acid	2.26	1.22
	Spermidine	2.3	1.36
	Ouabain	2.33	1.13
	N-Acetyl-DL-glutamic acid	2.35	1.38
	Uric acid	2.37	1.11
	2-Hydroxy-2-methylbutanedioic acid	2.38	1.16
	L-Serine	2.4	1.41
	Taurine	2.44	1.68
	Xanthosine	2.51	1.3
	gamma-Glutamylleucine	2.56	1.41
	Aniline	2.59	2.06
	trans-Aconitic acid	2.76	1.1
	gamma-Glutamyltyrosine	2.82	1.43
	N,N-Dimethylarginine	2.85	1.41
	Imidazoleacetic acid	2.85	1.18
	S-Adenosylhomocysteine	2.99	1.42
	Thiamine Pyrophosphate	3.04	1.36
	Pyrrole-2-carboxylic acid	3.1	1.63
	P-Aminobenzoate	3.12	1.68
	L-Dopa	3.13	1.59
	N1-Acetylspermine	3.25	1.58
	Adenosine	3.25	1.48
	Citrulline	3.28	1.28
	Proline-hydroxyproline	3.42	1.61
	Bilirubin	3.58	1.38
	Phenylpyruvic acid	4.07	1.72
	6-Methylnicotinamide	4.21	2.3
	Lignoceric acid	4.24	2.75
	Ascorbic acid	4.63	1.31
	L-Kynurenine	5.04	1.69
	L-Ascorbate	6.23	1.53
	3-Phenyllactic acid	8.14	1.55
	Glutaconic acid	9.49	1.15

FC, fold change; VIP, Variable Importance for the Projection.

**Table 2:** Microbial-related metabolites may be involved in eight major metabolism-based biological processes in GC.

Microbial-Related Metabolic Functions	Metabolites Enriched Pathways
ko00230	Purine metabolism
ko00730	Thiamine metabolism
ko00270	Cysteine and methionine metabolism
ko00240	Pyrimidine metabolism
ko00600	Sphingolipid metabolism
ko00010	Glycolysis/Gluconeogenesis
ko00970	Aminoacyl-tRNA biosynthesis
ko00520	Amino sugar and nucleotide sugar metabolism

## Data Availability

The datasets generated and analyzed during the study are available from the corresponding author on reasonable request.

## Supplementary Materials

The following supporting information can be downloaded at: https://www.mdpi.com/article/10.3390/cancers15215271/s1. Figure S1: PCA and PLA-DA analysis of metabolites in GC tissues and NC tissues. (A) PCA analysis showed an overall metabolites difference between GC tissues and NC tissues. (B) PLS-DA analysis showed the model was not over-fitting. Figure S2: The overall microbe composition of GC tissues and NC tissues. Figure S3: The differences of α diversity between GC tissues and NC tissues. Figure S4: The differences of β diversity between GC tissues and NC tissues. (A) Unweighted UniFrac distance metrics. (B) Jaccard_distance metrics. (C) Weighted UniFrac distance metrics. (D) Bray–Curtis_distance_matrix. Figure S5: The abundances of metabolites and microbes in each sample. (A) The abundances of the top 7 phyla and each metabolite in each sample. (B) The abundances of the top 7 phyla and each genus in each sample. Figure S6. Microbial-related metabolic function prediction by PICRUSt2. Table S1: General information of 30 GC patients. Table S2: Differential metabolites between GC tissues and NC tissues by T test. Table S3: Differential microbe in phyla and genera by *t*-test. Table S4: The coexistence probability of each differential metabolite and microbe.
