# Supplementary material for: Microbial-Related Metabolites May Be Involved in Eight Major Biological Processes and Represent Potential Diagnostic Markers in Gastric Cancer
Source: Cancers (Basel). 2023 Nov 3;15(21):5271. doi: 10.3390/cancers15215271 (PMC10649575; doi:10.3390/cancers15215271)
Supplement: Supplementary file 1 [file cancers-15-05271-s001.zip › Supplemental Material - cancers/Supplemental Material captions.docx]

Table S1. General information of 30 GC patients.

Table S2. Differential metabolites between GC and NC tissues by T test.

Table S3. Differential microbe in phyla and genera by T test.

Table S4. The coexistence probability of each differential metabolite and microbe.

**Figure S1.** PCA and PLA-DA analysis of metabolites in GC and NC tissues. (A) PCA analysis showed an overall metabolites difference between GC and NC tissues. (B) PLS-DA analysis showed the model was not over-fitting.

**Figure S2.** The overall microbe composition of GC and NC tissues.

**Figure S3.** The differences of α diversity between GC and NC tissues

**Figure S4.** The differences of β diversity between GC and NC tissues. (A) Unweighted-unifrac distance metrics. (B) Jaccard_distance metrics. (C) Weighted-unifrac distance metrics. (D) Bray-curtis_distance_matrix.

**Figure S5.** The abundances of metabolites and microbes in each sample. (A) The abundances of the top 7 phyla and each metabolite in each sample. (B) The abundances of the top 7 phyla and each genus in each sample.

**Figure S6.** Microbial-related metabolic function prediction by PICRUSt2.
