# Supplementary material for: Microbial-Related Metabolites May Be Involved in Eight Major Biological Processes and Represent Potential Diagnostic Markers in Gastric Cancer
Source: Cancers (Basel). 2023 Nov 3;15(21):5271. doi: 10.3390/cancers15215271 (PMC10649575; doi:10.3390/cancers15215271)
Supplement: Supplementary file 1 [file cancers-15-05271-s001.zip › Supplemental Material - cancers/Table S1.docx]

Table S1. General information of 30 GC patients.

| **Characteristics** | **Number (%)** |
| --- | --- |
| **Age(years)** |  |
| < 60 | 10 (33.3%) |
| ≥60 | 20 (33.3%) |
| **Gender** |  |
| Female | 8 (26.7%) |
| Male | 22 (73.3%) |
| **Smoking** |  |
| Yes | 15 (50.0%) |
| No | 15 (50.0%) |
| **Drinking** |  |
| Yes | 13 (43.3%) |
| No | 17 (56.7%) |
