# Supplementary material for: Microbial-Related Metabolites May Be Involved in Eight Major Biological Processes and Represent Potential Diagnostic Markers in Gastric Cancer
Source: Cancers (Basel). 2023 Nov 3;15(21):5271. doi: 10.3390/cancers15215271 (PMC10649575; doi:10.3390/cancers15215271)
Supplement: Supplementary file 1 [file cancers-15-05271-s001.zip › Supplemental Material - cancers/Table S2.docx]

| Table S2. Differential metabolites between GC and NC tissues by T test. | | | | | | |
| --- | --- | --- | --- | --- | --- | --- |
| Metabolites | NC.MeanSD | GC.MeanSD | NC.MedianIQR | GC.MedianIQR | FC | P |
| 6-Methylnicotinamide | 14.3 (9.89) | 75.87 (52.74) | 13.19 [8.364,17.168] | 55.52 [35.622,113.829] | 4.21 | 0 |
| Aniline | 72.62 (18.76) | 225.14 (154.22) | 68.85 [62.321,81.4] | 177.99 [112.258,260.064] | 2.59 | 0 |
| Oleic acid | 930.15 (530.72) | 234.26 (130.17) | 981.74 [398.847,1462.279] | 218.9 [142.076,262.844] | 0.22 | 0 |
| Lignoceric acid | 0.64 (0.26) | 2.49 (1.41) | 0.57 [0.511,0.64] | 2.43 [1.402,3.685] | 4.24 | 0 |
| Punicic Acid | 167.62 (123.04) | 36.71 (20.88) | 119.64 [56.125,260.536] | 29.56 [22.833,42.647] | 0.25 | 0 |
| 10E,12Z-Octadecadienoic acid | 23.98 (46.32) | 2.24 (0.73) | 5.36 [3.416,9.403] | 2.19 [1.842,2.574] | 0.41 | 0 |
| Methyl palmitate | 20.85 (13.98) | 4.61 (2.24) | 17.7 [11.436,29.267] | 4.34 [3.258,5.408] | 0.25 | 0 |
| 3-Ketodihydrosphingosine | 13.38 (5.5) | 5.14 (2.5) | 13.51 [11.105,15.95] | 4.91 [3.289,6.143] | 0.36 | 0 |
| L-Kynurenine | 35.24 (15.62) | 474.38 (613.04) | 36.15 [31.401,41.213] | 182.23 [83.254,538.836] | 5.04 | 0 |
| 5'-S-Methyl-5'-thioadenosine | 0.83 (0.23) | 2.43 (3.04) | 0.8 [0.65,0.953] | 1.36 [1.101,2.387] | 1.69 | 0 |
| Sorbitan monostearate | 12.03 (17.75) | 1.31 (1) | 5.95 [2.987,12.179] | 1.07 [0.564,1.542] | 0.18 | 0 |
| Phenylpyruvic acid | 40.24 (48.98) | 194.75 (272.78) | 22.81 [18.612,46.587] | 92.93 [76.396,139.369] | 4.07 | 0 |
| 8Z,11Z,14Z-Eicosatrienoic acid | 23156.54 (9239.64) | 39777.92 (12355.95) | 20923.94 [16465.126,24922.962] | 36987.56 [33033.122,46571.916] | 1.77 | 0 |
| Lauric acid | 16382.64 (14663.62) | 3633.32 (2771.38) | 11381.26 [5386.173,27009.751] | 2726.32 [2286.453,4193.732] | 0.24 | 0 |
| Menaquinone | 22.79 (59.87) | 1.51 (1.33) | 9.61 [2.332,16.382] | 1.3 [1.086,1.444] | 0.14 | 0 |
| dihydrotachysterol | 16.48 (16.65) | 3.24 (2.75) | 12.45 [8,17.724] | 1.98 [1.349,4.105] | 0.16 | 0 |
| Monoolein | 7254.44 (9047.1) | 377.62 (994.22) | 3544.99 [1278.988,11740.387] | 131.46 [86.338,303.052] | 0.04 | 0 |
| Taurine | 204.38 (105.54) | 468.97 (208.14) | 181.82 [134.202,251.637] | 442.86 [323.792,609.679] | 2.44 | 0 |
| DL-methionine sulfoxide | 105.65 (34.3) | 190.55 (70.2) | 105.28 [87.168,129.546] | 185.9 [140.978,223.913] | 1.77 | 0 |
| Pyrrole-2-carboxylic acid | 8.13 (4.68) | 26.87 (17.51) | 7.16 [5.168,10.683] | 22.18 [15.846,37.971] | 3.1 | 0 |
| Phosphocholine | 80.94 (31.16) | 44.94 (17.61) | 81.26 [55.947,91.075] | 41.63 [33.748,61.374] | 0.51 | 0 |
| 1-Palmitoylglycerol | 10763.21 (10276.2) | 1416.38 (1488.22) | 7710.75 [3061.835,14961.082] | 862.79 [440.727,1964.699] | 0.11 | 0 |
| Propionyl-L-carnitine | 4.72 (3) | 1.9 (0.99) | 4.33 [2.353,6.025] | 1.56 [1.271,2.403] | 0.36 | 0 |
| L-Dopa | 1.08 (0.66) | 3.67 (2.38) | 0.97 [0.598,1.482] | 3.05 [1.941,5.289] | 3.13 | 0 |
| P-Aminobenzoate | 10.29 (5.49) | 48.97 (56.89) | 9.21 [6.162,11.996] | 28.69 [15.019,58.051] | 3.12 | 0 |
| Thiamine Pyrophosphate | 1.98 (1.55) | 5.09 (2.54) | 1.71 [0.541,3.049] | 5.21 [3.741,6.083] | 3.04 | 0 |
| 7-Ketocholesterol | 61.45 (33.91) | 24.05 (28.91) | 53.73 [35.665,88.927] | 17.07 [11.396,28.88] | 0.32 | 0 |
| N1-Acetylspermine | 4.05 (5.11) | 20.78 (33.93) | 2.54 [2.005,3.826] | 8.26 [4.372,22.409] | 3.25 | 0 |
| cis-2-Decenoic acid | 1246.15 (1366.53) | 216.5 (241.29) | 859.06 [248.49,1624.304] | 151.31 [82.399,219.133] | 0.18 | 0 |
| Docosapentaenoic acid | 14760.87 (6716.44) | 24776.01 (7104.66) | 12663.8 [11224.455,18233.676] | 26054.97 [18876.479,29350.588] | 2.06 | 0 |
| Adrenic acid | 12506.41 (6614.27) | 24144.57 (10874.52) | 10193.3 [8704.969,12943.674] | 21481.02 [17195.255,32264.646] | 2.11 | 0 |
| Proline-hydroxyproline | 5.35 (12.09) | 14.2 (20.9) | 2.33 [1.659,2.943] | 7.99 [4.709,12.122] | 3.42 | 0 |
| Cystine | 17.68 (5.28) | 34.55 (24.03) | 16.76 [14.567,21.99] | 31.93 [23.813,37.237] | 1.91 | 0 |
| 3-Phenyllactic acid | 19.39 (30.15) | 182.5 (407.94) | 7.38 [4.85,21.793] | 60.09 [20.438,179.899] | 8.14 | 0 |
| S-Sulfo-L-Cysteine | 0.12 (0.07) | 0.18 (0.06) | 0.11 [0.095,0.13] | 0.17 [0.136,0.206] | 1.46 | 0 |
| N-Acetylmannosamine | 38.01 (15.47) | 77.78 (35.78) | 35.8 [30.71,41.236] | 72.39 [52.017,101.554] | 2.02 | 0 |
| Jasmonic acid | 11.25 (11.35) | 3.98 (2) | 7.43 [4.821,13.089] | 3.24 [2.723,4.687] | 0.44 | 0 |
| N-Acetyl-DL-glutamic acid | 4.43 (2.25) | 11.24 (7.67) | 3.96 [3.001,5.246] | 9.33 [6.408,14.638] | 2.35 | 0 |
| Muscone | 107.95 (115.27) | 17.97 (11.83) | 56.45 [25.739,143.106] | 14.42 [11.165,18.309] | 0.26 | 0 |
| gamma-Glutamylleucine | 138.42 (94.47) | 365.44 (248.16) | 107.83 [79.3,168.567] | 276.37 [194.226,473.733] | 2.56 | 0 |
| Palmitoleic acid | 58.31 (53.26) | 5.54 (8.25) | 51.42 [6.768,89.372] | 3.38 [2.868,4.257] | 0.07 | 0 |
| Valeric acid | 67.25 (20) | 154.86 (249.1) | 66.66 [56.172,78.87] | 91.61 [77.346,105.931] | 1.37 | 0 |
| gamma-Glutamyltyrosine | 10.94 (7.22) | 33.44 (29.94) | 9.08 [6.055,14.988] | 25.64 [16.405,38.85] | 2.82 | 0 |
| 2'-Deoxyadenosine | 15.23 (19.63) | 30.06 (25.71) | 11.14 [8.608,13.813] | 22.2 [15.238,33.055] | 1.99 | 0 |
| p-Mentha-1,3,8-triene | 88.27 (58.4) | 46.68 (17.34) | 67.56 [52.154,91.45] | 40.98 [34.33,53.619] | 0.61 | 0 |
| trans-Aconitic acid | 38.46 (200.81) | 9.67 (9.78) | 1.35 [1.234,1.551] | 3.74 [1.699,16.153] | 2.76 | 0 |
| Adenosine | 0.32 (0.26) | 0.84 (0.83) | 0.21 [0.177,0.336] | 0.68 [0.371,1.016] | 3.25 | 0 |
| 1-Stearoylglycerol | 3382.94 (3144.19) | 1037.97 (672.81) | 2403.71 [1208.147,4524.113] | 922.67 [561.094,1288.856] | 0.38 | 0 |
| Glycerol | 19.79 (10.03) | 11.11 (2.91) | 18.5 [12.871,22.388] | 10.68 [9.164,12.074] | 0.58 | 0 |
| Thr-Leu | 8.96 (5.37) | 4 (3.05) | 7.5 [5.319,10.986] | 3.18 [1.725,5.272] | 0.42 | 0 |
| N,N-Dimethylarginine | 9.47 (7.24) | 26.94 (20.08) | 7.69 [4.421,10.498] | 21.94 [13.945,32.372] | 2.85 | 0 |
| Caffeine | 14.87 (48.32) | 24.22 (79.26) | 5.55 [5.247,6.709] | 9.03 [7.614,12.339] | 1.63 | 0 |
| Prostaglandin B2 | 9.46 (3.97) | 5.5 (2.11) | 9.11 [6.289,12.379] | 5.11 [4.007,6.556] | 0.56 | 0 |
| N-Acetylneuraminic acid | 9.09 (7.69) | 22.67 (21.24) | 6.6 [5.173,8.84] | 13.59 [9.513,26.819] | 2.06 | 0 |
| Spermidine | 674 (508.7) | 1279.58 (631.76) | 524.71 [373.785,793.948] | 1207.68 [875.864,1622.488] | 2.3 | 0 |
| N8-Acetylspermidine | 27.44 (16.06) | 69.39 (50.73) | 25.48 [14.847,34.663] | 51.51 [30.995,93.492] | 2.02 | 0 |
| L-Ascorbate | 3.63 (4.59) | 24.11 (24.18) | 2.41 [2.006,2.894] | 14.99 [3.276,39.985] | 6.23 | 0 |
| 3-Hydroxylidocaine | 3.87 (3.12) | 27.34 (144.54) | 3.46 [1.178,6.155] | 0.69 [0.568,1.032] | 0.2 | 0 |
| 4-Methylphenol | 7.5 (3.42) | 20.79 (30.31) | 6.81 [5.47,7.826] | 12.46 [8.177,19.419] | 1.83 | 0 |
| L-Hydroxyproline | 6.31 (11.17) | 8.98 (8.31) | 3.49 [2.448,4.697] | 6.36 [5.259,10.172] | 1.82 | 0 |
| Xanthosine | 5.42 (3.55) | 11.59 (5.97) | 4.75 [2.897,7.368] | 11.91 [7.671,15.713] | 2.51 | 0 |
| 9,10-Dihome | 111.7 (73.21) | 63.54 (49.53) | 94.83 [59.013,129.308] | 51.78 [42.577,64.302] | 0.55 | 0 |
| S-Adenosylhomocysteine | 0.89 (0.66) | 6.93 (13.41) | 0.67 [0.525,0.998] | 2.01 [0.946,3.713] | 2.99 | 0 |
| 2-Methylbutyroylcarnitine | 26.39 (33.44) | 9.14 (4.57) | 15.13 [9.352,26.448] | 7.75 [6.132,9.843] | 0.51 | 0 |
| Uridine 5'-diphosphoglucuronic acid | 1.72 (1.67) | 6.06 (11.31) | 1.2 [0.883,1.533] | 2.2 [1.767,2.792] | 1.83 | 0 |
| Docosatrienoic acid | 110.51 (65.04) | 55.62 (41.33) | 93.41 [59.311,137.613] | 37.84 [29.343,66.807] | 0.41 | 0.0001 |
| Carvone | 68.09 (115.71) | 21.82 (8.38) | 30.06 [22.562,39.861] | 18.17 [16.276,25.374] | 0.6 | 0.0001 |
| Alternariol | 2.87 (2.48) | 1.63 (1.05) | 2.05 [1.71,2.869] | 1.43 [1.09,1.745] | 0.7 | 0.0001 |
| Methyl dihydrojasmonate | 156.12 (149.15) | 82.37 (31.88) | 114.82 [94.328,156.657] | 75.48 [64.868,87.536] | 0.66 | 0.0001 |
| L-Glutamic acid | 1524.88 (614.1) | 2570.62 (1175.85) | 1439.02 [1127.546,1842.382] | 2489.92 [1839.206,3226.609] | 1.73 | 0.0001 |
| 19-Nortestosterone | 14.56 (11.95) | 8.27 (2.96) | 11.19 [9.351,13.872] | 7.85 [6.395,9.601] | 0.7 | 0.0001 |
| N-Acetylcysteine | 0.37 (0.14) | 0.57 (0.24) | 0.33 [0.292,0.378] | 0.52 [0.398,0.716] | 1.56 | 0.0001 |
| Trigonelline | 22.28 (9.31) | 38.49 (16.81) | 20.53 [17.591,25.453] | 39.69 [24.731,47.68] | 1.93 | 0.0001 |
| Docosahexaenoic acid | 39566.09 (23422.87) | 59546.99 (18486.85) | 29733.61 [23875.545,46372.07] | 58894.86 [47818.208,69673.54] | 1.98 | 0.0001 |
| Testosterone acetate | 22.72 (34.06) | 8.11 (16.7) | 8.41 [3.393,20.192] | 2.51 [1.514,4.01] | 0.3 | 0.0001 |
| L-Pyroglutamic acid | 769.65 (394.17) | 1278.64 (510.28) | 766.29 [466.949,1018.774] | 1240.72 [880.309,1595.52] | 1.62 | 0.0002 |
| Ouabain | 18.07 (18.44) | 37.35 (24.06) | 14.68 [5.421,22.251] | 34.14 [19.591,46.935] | 2.33 | 0.0002 |
| N-Acetyl-L-leucine | 6.94 (6.31) | 11.06 (13.06) | 5.26 [4.267,6.275] | 7.04 [5.921,9.333] | 1.34 | 0.0002 |
| L-Serine | 88.85 (48.4) | 185.15 (121.38) | 73.16 [61.809,106.202] | 175.32 [110.472,224.769] | 2.4 | 0.0002 |
| Lauric acid ethyl ester | 48434.19 (36168.62) | 15623.26 (8841.4) | 44207.01 [14324.476,74044.536] | 15113.06 [9174.804,18482.101] | 0.34 | 0.0002 |
| Biotin | 4.46 (3.65) | 1.81 (0.82) | 3.58 [1.746,6.411] | 1.6 [1.353,1.744] | 0.45 | 0.0002 |
| Celestolide | 114.19 (67.94) | 57.09 (51.13) | 99.4 [69.146,155.69] | 46.74 [18.678,81.914] | 0.47 | 0.0003 |
| Targinine | 16.8 (8.72) | 30.23 (16.45) | 16 [10.866,19.883] | 28.92 [17.281,40.017] | 1.81 | 0.0003 |
| Feruloylcholine | 7.38 (4.28) | 3.65 (2.19) | 7.33 [3.672,9.537] | 3.19 [2.25,4.467] | 0.44 | 0.0003 |
| Corchorifatty acid F | 190.35 (356.45) | 47.62 (42.66) | 59.82 [45.235,115.684] | 33.64 [24.192,49.758] | 0.56 | 0.0003 |
| Flavin adenine dinucleotide | 12.74 (7.43) | 7.31 (3.48) | 10.86 [7.159,15.102] | 6.78 [5.283,8.003] | 0.62 | 0.0004 |
| 2-Hydroxy-2-methylbutanedioic acid | 36.78 (96.79) | 52.13 (38.26) | 18.49 [11.617,24.401] | 43.97 [21.016,68.523] | 2.38 | 0.0004 |
| Citrulline | 7.96 (14.13) | 27.79 (42.09) | 5.07 [2.852,7.998] | 16.6 [6.744,23.434] | 3.28 | 0.0004 |
| Bilirubin | 2.4 (3.55) | 4.98 (5.19) | 1.13 [0.764,2.508] | 4.04 [2.027,6.307] | 3.58 | 0.0005 |
| Glycerol 1-hexadecanoate | 90.96 (55.9) | 139.16 (66.34) | 75 [60.677,104.095] | 128.92 [93.818,178.777] | 1.72 | 0.0005 |
| Xanthine | 3022.38 (3927.31) | 4524.01 (2721.35) | 1906.95 [1224.934,3346.541] | 4182.32 [2537.447,5380.135] | 2.19 | 0.0005 |
| 2'-Deoxyinosine | 4.76 (13.38) | 11.71 (27.48) | 1.13 [0.73,1.597] | 2.25 [1.509,5.453] | 2 | 0.0007 |
| 7,8-Dihydroneopterin | 3.66 (2.48) | 6.8 (3.99) | 3.21 [1.294,5.422] | 6.29 [3.47,9.117] | 1.96 | 0.0007 |
| Ascorbic acid | 89.55 (124.41) | 479.86 (577.11) | 57.28 [47.794,82.566] | 265.18 [63.553,721.235] | 4.63 | 0.0008 |
| 2-Hydroxyvaleric acid | 39.16 (22.65) | 74.88 (54.99) | 34.72 [26.343,50.015] | 58.45 [41.526,100.623] | 1.68 | 0.0008 |
| Uric acid | 140 (113.57) | 290.13 (207.38) | 114.65 [62.41,172.255] | 271.89 [139.929,335.78] | 2.37 | 0.0008 |
| 3-Coumaric acid | 16.31 (7.78) | 29.49 (17.04) | 14.96 [12.217,18.803] | 27.15 [16.616,39.526] | 1.82 | 0.0008 |
| N-Acetylalanine | 94.26 (39.39) | 163.54 (84.01) | 93.41 [72.42,116.788] | 164 [94.342,209.572] | 1.76 | 0.0009 |
| L-Cystine | 34.58 (12.02) | 56.7 (28.56) | 33.07 [27.686,42.387] | 58.16 [35.482,71.804] | 1.76 | 0.0009 |
| Spermine | 201.49 (540.53) | 183.37 (162.82) | 66.04 [48.615,96.537] | 140.62 [87.97,187.531] | 2.13 | 0.0009 |
| Putrescine | 83.72 (61.67) | 242.3 (482.27) | 74.11 [48.713,91.819] | 131.17 [74.487,204.39] | 1.77 | 0.001 |
| DL-Mandelic acid | 3.62 (4.96) | 8.32 (20.18) | 2.54 [2.234,2.971] | 3.26 [2.666,4.52] | 1.28 | 0.001 |
| Glutaconic acid | 62.71 (182.81) | 351.59 (589.78) | 6.48 [5.12,12.316] | 61.51 [8.48,511.673] | 9.49 | 0.0011 |
| Undecanoic acid | 55.73 (43.69) | 43.32 (70.95) | 40.29 [26.55,62.093] | 22.91 [20.16,32.238] | 0.57 | 0.0012 |
| mesaconic acid | 68.8 (269.59) | 52.68 (40.27) | 16.27 [10.926,27.331] | 44.4 [21.53,76.448] | 2.73 | 0.0013 |
| cis-gondoic acid | 15.95 (18.08) | 8.15 (2.61) | 10.41 [8.107,14.244] | 8.12 [6.973,9.311] | 0.78 | 0.0013 |
| Glycyl-L-Proline | 3.91 (2.13) | 11.78 (17.49) | 3.18 [2.534,4.421] | 6.11 [3.303,10.957] | 1.92 | 0.0016 |
| Hydroxypyruvic acid | 3.64 (2.18) | 5.37 (2.7) | 3.41 [2.109,4.283] | 4.76 [3.596,6.948] | 1.4 | 0.0016 |
| Estradiol | 4.33 (3.18) | 4.99 (13.16) | 3.6 [1.961,5.991] | 1.47 [1.239,2.699] | 0.41 | 0.0017 |
| Creatinine | 129.3 (46.75) | 175.09 (61.46) | 126.82 [110.864,148.755] | 198.47 [133.717,205.471] | 1.56 | 0.0018 |
| Prostaglandin B1 | 95.2 (63.18) | 59.66 (60.96) | 83.93 [56.235,116.917] | 46.45 [23.652,65.755] | 0.55 | 0.0018 |
| Succinic semialdehyde | 140.45 (43.44) | 259.81 (269.2) | 133.94 [109.003,162.911] | 183.96 [154.751,252.724] | 1.37 | 0.0019 |
| Orotic Acid | 2.27 (2.96) | 4.21 (4.52) | 0.8 [0.709,1.919] | 1.81 [0.985,5.847] | 2.26 | 0.002 |
| Coumarin | 102.89 (34.5) | 142.88 (54.74) | 99.39 [84.379,115.3] | 147.12 [105.073,162.977] | 1.48 | 0.0021 |
| Dihydrouracil | 119.89 (66.56) | 216.7 (137.41) | 100.27 [73.548,163.609] | 172.54 [116.903,315.239] | 1.72 | 0.0021 |
| Decanoic acid | 5824.21 (6063.96) | 3306.07 (4730.09) | 4530.56 [2329.292,6100.924] | 1422.59 [881.697,3233.528] | 0.31 | 0.0022 |
| Benzoic acid | 134.19 (144.67) | 217.76 (298.14) | 97.77 [81.743,126.558] | 124.94 [105.6,173.507] | 1.28 | 0.0022 |
| L-5-Hydroxytryptophan | 160.22 (265.77) | 70.34 (126.28) | 62.81 [36.373,107.325] | 27.36 [19.681,51.566] | 0.44 | 0.0025 |
| 3-Hydroxy-4-methoxyphenylacetic acid | 6.3 (3.64) | 9.48 (4.69) | 5.02 [4.176,7.581] | 8.37 [5.986,12.427] | 1.67 | 0.0026 |
| Hexadecanamide | 26381.42 (11867.1) | 18445.21 (10910.3) | 27782.55 [19022.197,32012.907] | 15844.1 [12535.254,21855.773] | 0.57 | 0.0026 |
| D-Alanyl-D-Alanine | 29.87 (19.17) | 41.2 (22.81) | 25.14 [21.484,29.766] | 36.61 [26.303,46.592] | 1.46 | 0.0029 |
| Kynurenic acid | 6.15 (6.56) | 38.37 (121.05) | 4.6 [3.928,5.314] | 6.37 [4.707,8.437] | 1.38 | 0.0029 |
| Stearamide | 11280.64 (7337.51) | 6339.31 (5900.5) | 10861.05 [5814.673,15767.372] | 4393.73 [2997.169,9502.238] | 0.4 | 0.0029 |
| beta hydroxybutyrate | 8.49 (6.21) | 3.83 (0.75) | 5.57 [3.582,12.042] | 3.71 [3.248,4.392] | 0.67 | 0.003 |
| Taurochenodesoxycholic Acid | 7.87 (2.98) | 6.42 (3.44) | 6.58 [5.772,10.444] | 5.23 [4.384,7.413] | 0.79 | 0.003 |
| Imidazoleacetic acid | 47.34 (106.53) | 79.25 (156.41) | 12.39 [10.709,25.23] | 35.33 [16.695,49.777] | 2.85 | 0.0035 |
| 6-Phosphogluconic acid | 4.78 (5.02) | 10.94 (11.37) | 3.87 [1.369,5.685] | 7.56 [3.709,11.72] | 1.96 | 0.0037 |
| 4-Hydroxyphenylpyruvic acid | 1.25 (1) | 1.52 (1.3) | 0.97 [0.836,1.181] | 1.18 [1.033,1.434] | 1.22 | 0.0037 |
| Isobutyric acid | 63.89 (78.86) | 441.8 (1440.74) | 41.18 [35.915,50.047] | 55.56 [45.124,74.392] | 1.35 | 0.0037 |
| trans-Cinnamic acid | 140.2 (88.29) | 269.46 (189.48) | 118.75 [89.074,152.973] | 212.16 [124.244,355.111] | 1.79 | 0.0039 |
| 2-Aminoethanesulfinic Acid | 0.86 (0.61) | 1.2 (0.54) | 0.59 [0.499,1.009] | 1.29 [0.675,1.739] | 2.17 | 0.0039 |
| N,N-Dimethylsphingosine | 202.57 (186.75) | 121.2 (155.16) | 148.02 [104.106,229.487] | 71.35 [39.028,129.552] | 0.48 | 0.0039 |
| (R)-Lipoic Acid | 22.73 (17.57) | 43.89 (36.36) | 19.38 [14.995,26.398] | 37.97 [22.827,51.649] | 1.96 | 0.0041 |
| 3-Hydroxybutyric acid | 278.19 (241.16) | 566.95 (513.17) | 191.91 [125.33,366.205] | 373.6 [244.798,711.176] | 1.95 | 0.0041 |
| Sn-Glycero-3-Phosphocholine | 4.19 (1.94) | 3.15 (0.96) | 3.58 [3.134,4.32] | 2.93 [2.397,3.733] | 0.82 | 0.0043 |
| N-Acetylglycine | 8.58 (10.22) | 21.65 (34.19) | 6.21 [5.274,7.303] | 7.86 [6.538,15.398] | 1.27 | 0.005 |
| DL-Malic acid | 346.64 (201.32) | 707.96 (649.37) | 302.27 [200.565,478.029] | 487.33 [354.064,797.517] | 1.61 | 0.0055 |
| D-Saccharic acid | 121 (291.2) | 126.65 (118.62) | 38.19 [29.291,61.744] | 102.77 [48.019,153.31] | 2.69 | 0.0055 |
| 6-Pentyl-2H-pyran-2-one | 11.54 (8.5) | 7.72 (2.38) | 8.97 [7.54,11.897] | 7.63 [5.967,8.818] | 0.85 | 0.0055 |
| Citric acid | 99.53 (227.72) | 122.51 (122.1) | 34.71 [26.669,58.947] | 98.58 [41.3,146.743] | 2.84 | 0.0058 |
| 2-Phenylacetamide | 10.9 (4.87) | 16.22 (9.13) | 9.54 [7.953,11.784] | 12.34 [9.191,21.533] | 1.29 | 0.0061 |
| 1-Phenyl-3-methyl-5-pyrazolone | 21.95 (29.23) | 11.74 (12.1) | 12.3 [8.574,15.734] | 7.47 [5.964,11.986] | 0.61 | 0.0061 |
| Indole-3-lactic acid | 20.33 (11.8) | 35.83 (29.09) | 16.79 [13.499,24.308] | 27.11 [17.591,38.568] | 1.61 | 0.0063 |
| Palmitoyl ethanolamide | 751.96 (510.17) | 442.85 (360.33) | 664.56 [446.189,1074.173] | 307.26 [183.428,610.558] | 0.46 | 0.0063 |
| L-Ergothioneine | 6.14 (2.69) | 13.11 (12.77) | 5.6 [4.476,7.531] | 8.49 [5.334,16.586] | 1.52 | 0.0067 |
| Tridecylic acid | 409.04 (349.72) | 230.62 (137.89) | 335.6 [189.202,489.155] | 185.74 [159.341,231.814] | 0.55 | 0.0067 |
| Deoxyadenosine | 80.28 (30.55) | 107.95 (38.41) | 82.09 [63.587,94.976] | 102.48 [82.486,133.352] | 1.25 | 0.007 |
| Cytidine 5'-diphosphocholine | 2.98 (2.37) | 7.94 (12.84) | 2.24 [1.865,2.834] | 3.11 [2.298,8.471] | 1.39 | 0.007 |
| 17alpha-Hydroxyprogesterone | 5.48 (3.48) | 3.38 (1.52) | 4.22 [3.259,6.743] | 3.18 [2.457,4.254] | 0.75 | 0.0076 |
| Adrenosterone | 5.28 (5.92) | 2.83 (2.77) | 3.6 [1.991,6.093] | 2.14 [1.616,2.839] | 0.59 | 0.008 |
| 5-Hydroxy-L-Tryptophan | 16.39 (17.8) | 10.69 (10.76) | 9.68 [7.448,17.35] | 6.02 [4.535,9.559] | 0.62 | 0.0084 |
| Chrysin | 4.56 (2.25) | 6.54 (5.97) | 3.72 [3.311,4.54] | 5.57 [3.989,6.535] | 1.5 | 0.0088 |
| 6-phospho-D-glucono-1,5-lactone | 1.97 (1.19) | 2.9 (1.7) | 1.88 [1.251,2.288] | 2.56 [1.704,3.752] | 1.36 | 0.0088 |
| Progesterone | 8.96 (10.78) | 4.68 (4.53) | 5.49 [3.328,9.206] | 3.18 [2.353,4.444] | 0.58 | 0.0088 |
| 1,3-Dimethyluracil | 125.37 (104.24) | 76.8 (103.59) | 80.49 [49.912,180.35] | 38.25 [28.061,68.416] | 0.48 | 0.0096 |
| o-Cresol | 472.01 (340.3) | 302.41 (148.31) | 373.56 [277.383,440.004] | 266.46 [208.773,367.495] | 0.71 | 0.0096 |
| L-(-)-Methionine | 4019.84 (2557.47) | 6609.41 (4623.81) | 3432.46 [2244.356,5166.055] | 6546.9 [3251.316,8006.402] | 1.91 | 0.01 |
| Triptolide | 32.66 (15.5) | 22.9 (14.7) | 34.66 [19.065,44.131] | 20.1 [12.885,27.951] | 0.58 | 0.01 |
| 2-Hydroxycaproic acid | 22.11 (11.71) | 61.73 (104.75) | 19.73 [15.449,26.86] | 25.22 [20.08,48.414] | 1.28 | 0.011 |
| Acetylcarnitine | 205.8 (177.18) | 425.17 (408.74) | 152.37 [100.742,222.911] | 242.67 [160.135,607.999] | 1.59 | 0.011 |
| Epitestosterone | 75.7 (97.28) | 29.91 (32.01) | 39.07 [21.524,65.728] | 22.52 [12.261,38.749] | 0.58 | 0.011 |
| 16-Hydroxyhexadecanoic acid | 96110.28 (64313.74) | 54194.06 (30617.17) | 63757.95 [43194.297,145842.155] | 47816.04 [32320.128,64744.392] | 0.75 | 0.011 |
| 4-Pyridoxic acid | 5.47 (3.32) | 3.99 (1.66) | 4.47 [3.932,5.487] | 3.52 [2.787,5.035] | 0.79 | 0.011 |
| A-Ketoglutaric Acid | 12.14 (60.74) | 1.25 (0.29) | 1.09 [0.918,1.194] | 1.22 [1.074,1.459] | 1.11 | 0.011 |
| D-Glucose 6-phosphate | 8.05 (4.33) | 11.72 (6.09) | 6.43 [4.348,11.389] | 12.18 [6.809,14.79] | 1.89 | 0.012 |
| Cafestol | 143.99 (107.54) | 90.95 (91.56) | 111.63 [61.535,200.054] | 61.98 [29.512,95.478] | 0.56 | 0.012 |
| L-Tyrosine | 6431.15 (3169.15) | 9638.99 (5150.11) | 6009.55 [4383.288,7985.512] | 8107.72 [6251.144,13094.586] | 1.35 | 0.013 |
| Pyrophosphate | 336.92 (112.53) | 401.13 (131.41) | 351.73 [291.119,380.124] | 412.01 [318.695,491.327] | 1.17 | 0.013 |
| Norharman | 71.17 (294.43) | 3.06 (5.44) | 1.85 [1.086,3.07] | 1.03 [0.755,1.841] | 0.56 | 0.013 |
| T-2 Triol | 16.72 (18.72) | 10.34 (6.48) | 9.78 [8.743,11.984] | 7.96 [7.28,10.455] | 0.81 | 0.014 |
| N-Acetyl-L-phenylalanine | 3.51 (4.56) | 6.42 (6.15) | 2.11 [1.315,3.684] | 3.95 [2.391,9.443] | 1.87 | 0.015 |
| Mevalonic acid | 14.75 (13.38) | 25.53 (30.05) | 10.37 [7.792,14.863] | 18.88 [11.639,28.216] | 1.82 | 0.015 |
| Ala-trp | 7.22 (19.88) | 4.09 (5.19) | 2.89 [2.289,4.63] | 2.12 [1.555,3.47] | 0.74 | 0.015 |
| Avocadyne 1-acetate | 141.1 (121.7) | 79.19 (62.95) | 115.37 [60.537,177.519] | 59.23 [39.5,96.812] | 0.51 | 0.015 |
| Medroxyprogesterone | 243.67 (323.23) | 118.21 (160.8) | 108.3 [73.228,182.408] | 71.74 [48.003,121.866] | 0.66 | 0.015 |
| 4-Hydroxyphenylacetic acid | 3.8 (1.18) | 4.56 (1.89) | 3.57 [2.91,4.058] | 4.09 [3.763,4.758] | 1.14 | 0.016 |
| Glyoxylate | 5.4 (2.56) | 7.35 (3.25) | 4.89 [3.478,6.495] | 6.8 [5.021,9.893] | 1.39 | 0.016 |
| Methylimidazoleacetic acid | 32.98 (10.21) | 62.57 (100.9) | 31.24 [27.157,38.226] | 38.58 [32.017,43.198] | 1.23 | 0.016 |
| D-Sedoheptulose 7-phosphate | 24.35 (19.22) | 41.05 (30.06) | 18.66 [12.116,30.121] | 35.1 [17.986,53.401] | 1.88 | 0.016 |
| 3-Phosphoglyceric acid | 4.98 (3.19) | 3.75 (1.85) | 4.19 [3.196,5.66] | 3.17 [2.66,4.123] | 0.76 | 0.016 |
| Hexanoic acid | 144.51 (74.42) | 192.14 (483.27) | 124.96 [85.25,173.694] | 96.26 [64.042,111.013] | 0.77 | 0.017 |
| Glutamine | 4.06 (1.34) | 4.8 (1.38) | 3.56 [3.067,5.127] | 4.75 [3.652,5.756] | 1.33 | 0.018 |
| Valine | 3410.76 (1627.41) | 5023.24 (2662.8) | 2979.64 [2521.626,4185.405] | 4392.99 [2779.105,7259.317] | 1.47 | 0.018 |
| 2-Hydroxycinnamic acid | 6680.03 (3061.01) | 9826.59 (5305.27) | 6289.96 [4988.449,7870.876] | 8202.02 [6280.247,13088.811] | 1.3 | 0.018 |
| Nervonic acid | 150.1 (91.92) | 294.59 (340.87) | 129.71 [85.813,211.964] | 193.37 [136.64,342.601] | 1.49 | 0.018 |
| Thymine | 104.79 (225.92) | 159.43 (321.68) | 32.69 [23.783,54.757] | 67.88 [36.051,83.41] | 2.08 | 0.019 |
| Cotinine | 1.94 (3.59) | 4.67 (7.34) | 1.07 [0.909,1.457] | 1.91 [1.086,3.595] | 1.78 | 0.019 |
| N2,N2-Dimethylguanosine | 54.77 (46.37) | 80.46 (49.68) | 42.39 [21.509,78.271] | 77.88 [44.947,109.817] | 1.84 | 0.02 |
| Crotonic acid | 29.97 (99.32) | 21.94 (18.21) | 11.64 [8.484,14.384] | 16.14 [9.802,23.225] | 1.39 | 0.02 |
| Urethane | 133.9 (113.05) | 218.98 (179.09) | 110.87 [76.524,151.048] | 189.94 [87.244,281.691] | 1.71 | 0.021 |
| Ornithine | 220.75 (99.34) | 332.18 (180.61) | 215.42 [169.894,244.324] | 310.64 [193.954,405.339] | 1.44 | 0.021 |
| Tacrolimus | 73.03 (97.43) | 16.61 (34.65) | 17.44 [5.507,87.906] | 5.5 [3.997,15.381] | 0.32 | 0.021 |
| Proline | 4426.12 (2065.68) | 6015.44 (2750.31) | 4141.68 [3276.603,5098.817] | 5981.76 [3874.896,7080.262] | 1.44 | 0.022 |
| Aflatoxin M1 | 2.45 (1.38) | 3.37 (1.79) | 2.33 [1.801,2.941] | 3.52 [2.048,4.557] | 1.51 | 0.022 |
| D-Ribulose 5-phosphate | 52.05 (28.36) | 69.17 (35.62) | 48.27 [32.78,63.68] | 64.34 [44.963,80.425] | 1.33 | 0.023 |
| Acetophenone | 170.91 (37.29) | 210.04 (62.69) | 171.21 [153.989,194.361] | 197.59 [163.896,247.41] | 1.15 | 0.024 |
| Thymidine | 1.22 (3.97) | 1.15 (3.26) | 0.33 [0.244,0.384] | 0.26 [0.202,0.316] | 0.79 | 0.025 |
| 1-Methyladenosine | 23.71 (18.53) | 31.94 (16.7) | 17.39 [11.231,28.622] | 31.4 [21.081,41.273] | 1.81 | 0.026 |
| glutathione | 0.36 (0.39) | 0.78 (1.48) | 0.23 [0.206,0.296] | 0.3 [0.246,0.47] | 1.27 | 0.026 |
| D-Malic Acid | 14.71 (7.4) | 21.19 (12.55) | 13.38 [8.81,18.769] | 19.24 [11.626,26.075] | 1.44 | 0.027 |
| 2-(Formylamino)Benzoic Acid | 4.25 (1.07) | 6.28 (5.58) | 4.19 [3.535,4.729] | 4.65 [4.082,5.999] | 1.11 | 0.027 |
| D-Erythrose 4-phosphate | 27.36 (50.25) | 40.53 (48.57) | 11.97 [8.353,29.593] | 24.93 [15.47,43.326] | 2.08 | 0.028 |
| Adenosine 5'-monophosphate | 15.41 (7.23) | 25.06 (17.11) | 13.06 [10.738,21.323] | 24.79 [11.604,33.676] | 1.9 | 0.031 |
| 2-Deoxy-D-galactose | 151.78 (333.81) | 102.24 (69.17) | 46.84 [29.337,94.69] | 84.43 [52.371,142.779] | 1.8 | 0.031 |
| 5-Aminovaleric acid | 2042.58 (1644.85) | 3487.49 (2670.98) | 1689.36 [1079.443,2242.724] | 3022.21 [1565.978,5239.438] | 1.79 | 0.032 |
| Riboflavin | 87.6 (31.58) | 70.03 (27.7) | 81.65 [62.423,105.773] | 66.04 [50.953,87.712] | 0.81 | 0.033 |
| Methionine sulfoxide | 1712.72 (581.26) | 2043.24 (636.84) | 1756.64 [1382.827,2056.89] | 2074.87 [1630.373,2429.284] | 1.18 | 0.033 |
| Serine | 92.65 (44.78) | 128.97 (65.05) | 87.42 [66.586,111.029] | 132.15 [69.75,158.182] | 1.51 | 0.034 |
| Phenylacetylglutamine | 8.19 (8.73) | 17.68 (23.35) | 5.19 [1.759,9.204] | 7.63 [4.233,16.94] | 1.47 | 0.036 |
| L-Threonine | 659.98 (329.54) | 891.21 (461.47) | 598.81 [475.013,741.002] | 895.21 [570.156,1166.397] | 1.49 | 0.037 |
| Glycylproline | 21.86 (11.13) | 47.89 (64.86) | 20.2 [14.19,27.047] | 27.41 [17.823,44.737] | 1.36 | 0.037 |
| D-Cysteine | 7.37 (7.99) | 20.45 (31.71) | 3.75 [2.679,8.794] | 8.79 [3.055,21.402] | 2.34 | 0.037 |
| Thiamine | 2.5 (5.86) | 0.73 (0.75) | 0.65 [0.519,1.359] | 0.57 [0.49,0.718] | 0.87 | 0.037 |
| Ecgonine | 10.86 (14.4) | 6.22 (6.82) | 5.51 [4.12,10.442] | 3.83 [3.402,5.394] | 0.69 | 0.037 |
| Propylparaben | 3 (2.28) | 4.86 (12.19) | 2.12 [1.753,3.629] | 1.84 [1.461,2.454] | 0.87 | 0.037 |
| O-Desmethylnaproxen | 2.65 (1.15) | 3.4 (1.7) | 2.71 [2.035,3.134] | 3.6 [1.815,4.173] | 1.33 | 0.039 |
| Cysteine | 6.57 (6.7) | 19.43 (31.6) | 3.78 [2.799,7.364] | 7.87 [3.161,19.294] | 2.08 | 0.039 |
| 3-Acetyl-2,5-dimethylfuran | 8.38 (9.93) | 4.7 (2.31) | 5.21 [3.824,7.701] | 3.99 [3.645,4.725] | 0.77 | 0.039 |
| Phenobarbital | 3.54 (17.47) | 4.38 (21.61) | 0.33 [0.287,0.443] | 0.39 [0.35,0.484] | 1.2 | 0.039 |
| 1,5-Anhydro-D-glucitol | 39.33 (25.27) | 57.19 (36.16) | 37.8 [20.472,50.204] | 55.11 [30.819,73.668] | 1.46 | 0.04 |
| Threonine | 229.93 (106.26) | 316.9 (156.96) | 206.24 [169.785,276.536] | 323.8 [185.719,411.814] | 1.57 | 0.041 |
| 3-hydroxy-3-methylpentanedioic acid | 5.28 (3.22) | 7.39 (7.7) | 4.33 [3.516,5.516] | 5.05 [4.239,7.772] | 1.16 | 0.041 |
| Hypoxanthine | 4141.73 (2064.71) | 5380.88 (2536) | 4158.66 [2861.343,5689.395] | 5583.67 [3654.336,6826.41] | 1.34 | 0.041 |
| Kojic acid | 5.12 (11.15) | 4.08 (2.07) | 3.1 [2.284,3.71] | 3.7 [3.222,4.355] | 1.19 | 0.041 |
| 4-Hydroxybenzaldehyde | 536.37 (238.84) | 696.58 (316.44) | 525.84 [391.081,590.976] | 672.24 [443.09,890.153] | 1.28 | 0.043 |
| Neopterin | 6.1 (3.55) | 4.35 (2.05) | 4.51 [3.849,8.2] | 3.74 [2.88,5.562] | 0.83 | 0.043 |
| Cyclamic acid | 0.68 (1.51) | 0.89 (1.51) | 0.31 [0.274,0.387] | 0.39 [0.306,0.549] | 1.27 | 0.043 |
| Indole-3-acetic acid | 73.88 (173.25) | 354.42 (1162.13) | 24.12 [12.807,40.694] | 31.83 [25.958,67.636] | 1.32 | 0.045 |
| Mestranol | 504.56 (131.26) | 449.31 (231.8) | 464.07 [417.842,528.077] | 413.55 [333.394,503.76] | 0.89 | 0.046 |
| Leukotriene E4 | 10.77 (14.59) | 18.61 (20.85) | 4.24 [2.558,9.764] | 7.76 [3.819,37.213] | 1.83 | 0.046 |
