# Supplementary material for: Microbial-Related Metabolites May Be Involved in Eight Major Biological Processes and Represent Potential Diagnostic Markers in Gastric Cancer
Source: Cancers (Basel). 2023 Nov 3;15(21):5271. doi: 10.3390/cancers15215271 (PMC10649575; doi:10.3390/cancers15215271)
Supplement: Supplementary file 1 [file cancers-15-05271-s001.zip › Supplemental Material - cancers/Table S3.docx]

Table S3. Differential microbe in phyla and genera by T test.

| **Phylum** | **GC Mean (SD)** | **NC Mean (SD)** | **FC** | **FDR.P** |
| --- | --- | --- | --- | --- |
| *Thaumarchaeota* | 1937.28 (9974.7) | 3.16 (15.23) | 612.21 | 0.002 |
| *Acidobacteria* | 584.1 (2054.39) | 20.06 (56.37) | 29.12 | 0.025 |
| *Chloroflexi* | 73.55 (279.51) | 3.86 (19.06) | 19.04 | 0.013 |
| *Actinobacteria* | 6093.56 (21837.13) | 855.16 (2529.24) | 7.13 | 0.014 |
| *Firmicutes* | 86867.06 (183775.65) | 17603.31 (44054.71) | 4.93 | 0.002 |
| *Spirochaetes* | 85.73 (285.98) | 352.35 (1167.14) | 4.11 | 0.016 |
| *Bacteroidetes* | 28469.56 (107679.47) | 8211.5 (35552.52) | 3.47 | 0.014 |
| *Fusobacteria* | 963.01 (2212.65) | 389.41 (1618.33) | 2.47 | 0.018 |
| *Planctomycetes* | 10.63 (31.23) | 26.19 (60.34) | 2.46 | 0.014 |
| *Epsilonbacteraeota* | 18521.3 (90417.35) | 51352.02 (162238.85) | 0.36 | 0.011 |

| **Genus** | **GC Mean (SD)** | **NC Mean (SD)** | **FC** | **FDR.P** |
| --- | --- | --- | --- | --- |
| *Candidatus Nitrocosmicus* | 823.17 (4225.95) | 0.38 (0.07) | 2139.40 | 0.019 |
| *Candidatus Actinomarina* | 408.45 (2068.53) | 0.38 (0.07) | 1061.56 | 0.006 |
| *Prevotellaceae UCG-001* | 364.25 (1824.58) | 0.38 (0.07) | 946.69 | 0.016 |
| *uncultured Rubrobacteria bacterium* | 242.23 (1018.29) | 0.38 (0.07) | 629.55 | 0.012 |
| *Phreatobacter* | 164.02 (785.41) | 0.38 (0.07) | 426.28 | 0.012 |
| *Ezakiella* | 913.88 (4418.12) | 3.15 (10.74) | 289.69 | 0.002 |
| *Luteimonas* | 64.78 (312.05) | 0.38 (0.07) | 168.35 | 0.045 |
| *Flavonifractor* | 39.7 (128.43) | 0.38 (0.07) | 103.17 | 0.039 |
| *Rubrobacter* | 971.62 (4380.43) | 18.4 (38.04) | 52.79 | 0.008 |
| *Bacillus* | 28094.05 (146205.62) | 563.54 (1771.36) | 49.85 | 0.006 |
| *Ruminococcaceae UCG-014* | 3120.74 (14984.62) | 65.57 (244.93) | 47.60 | 0.005 |
| *Lachnoanaerobaculum* | 8866.36 (40733.94) | 215.62 (790.94) | 41.12 | 0.004 |
| *Lachnospiraceae UCG-010* | 12.14 (30.63) | 0.38 (0.07) | 31.54 | 0.039 |
| *Actinomyces* | 1235.6 (5762.31) | 50.59 (185.79) | 24.43 | 0.012 |
| *Lachnoclostridium* | 95.41 (292.75) | 5.96 (30.53) | 16.02 | 0.040 |
| *Solobacterium* | 315.07 (1184.55) | 23.2 (88.8) | 13.58 | 0.040 |
| *Cutibacterium* | 820.57 (3846.83) | 68.25 (308.82) | 12.02 | 0.009 |
| *Veillonella* | 1108.78 (4727.04) | 107.77 (461.78) | 10.29 | 0.028 |
| *Terrisporobacter* | 110.67 (236.14) | 10.85 (57.3) | 10.20 | 0.037 |
| *Stomatobaculum* | 863.84 (2977.35) | 137.64 (480.47) | 6.28 | 0.032 |
| *Lysinibacillus* | 364.95 (610.23) | 66.93 (235.16) | 5.45 | 0.009 |
| *Howardella* | 140.52 (550.77) | 31.28 (118.06) | 4.49 | 0.012 |
| *Prevotella 7* | 16582.76 (82755.01) | 3906.2 (18331.41) | 4.25 | 0.004 |
| *Campylobacter* | 125.88 (266.69) | 39.48 (155.75) | 3.19 | 0.039 |
| *Johnsonella* | 120.57 (318.36) | 40.86 (221.71) | 2.95 | 0.040 |
| *Prevotella* | 7806.85 (27263.71) | 2787.27 (13590.89) | 2.80 | 0.004 |
| *Fusobacterium* | 918.52 (2186.17) | 372.6 (1600.99) | 2.47 | 0.048 |
| *Eubacterium nodatum group* | 46.92 (131.68) | 22.02 (118.5) | 2.13 | 0.030 |
| *Parvimonas* | 872.34 (1730.55) | 436.46 (1474.41) | 2.00 | 0.011 |
| *Streptococcus* | 5593.75 (17403.46) | 3175.95 (11094.32) | 1.76 | 0.013 |
| *Gemella* | 339.22 (676.6) | 243.67 (924.22) | 1.39 | 0.012 |
| *Slackia* | 120.54 (417.69) | 90.94 (464.95) | 1.33 | 0.040 |
| *Serratia* | 82927.89 (105868.96) | 72717.98 (191059.67) | 1.14 | 0.004 |
| *Helicobacter* | 18394.33 (90341.05) | 51307.86 (162104.37) | 0.36 | 0.012 |
| *Niveispirillum* | 0.45 (0.14) | 11013.13 (45003.42) | 0.00 | 0.012 |
