# Supplementary material for: Microbial-Related Metabolites May Be Involved in Eight Major Biological Processes and Represent Potential Diagnostic Markers in Gastric Cancer
Source: Cancers (Basel). 2023 Nov 3;15(21):5271. doi: 10.3390/cancers15215271 (PMC10649575; doi:10.3390/cancers15215271)
Supplement: Supplementary file 1 [file cancers-15-05271-s001.zip › Supplemental Material - cancers/Table S4.docx]

| Table S4.The coexistence probability of each differential metabolite and phyla. | | | | | | | |
| --- | --- | --- | --- | --- | --- | --- | --- |
|  | *Thaumarchaeota* | *Acidobacteria* | *Actinobacteria* | *Bacteroidetes* | *Epsilonbacteraeota* | *Firmicutes* | *Fusobacteria* |
| 10E,12Z-Octadecadienoic acid | -0.3334 | 0.803293 | -2.28368 | -0.87465 | -1.44504 | -0.93281 | -0.55328 |
| 1-Palmitoylglycerol | -0.36342 | -1.24021 | 2.221219 | 0.664547 | 0.632832 | 0.639414 | 0.435145 |
| 1-Stearoylglycerol | -0.90894 | -0.30957 | 1.3713 | -0.31362 | 1.631668 | 0.106851 | -2.03603 |
| 2-Aminoethanesulfinic Acid | 1.26421 | -2.10588 | -2.62039 | -0.65319 | -1.97793 | -1.24815 | 2.013145 |
| 2'-Deoxyinosine | -1.3757 | 2.663385 | 1.860262 | -0.25668 | 3.360089 | 0.746083 | -4.24341 |
| 2-Hydroxy-2-methylbutanedioic acid | -0.50301 | 0.310374 | -0.79178 | -0.91256 | 0.614017 | -0.60494 | -1.94633 |
| 3-Hydroxylidocaine | -0.74627 | -2.20682 | -2.45564 | -1.10942 | -3.47355 | -1.67452 | 1.003111 |
| 3-Ketodihydrosphingosine | -2.57377 | 2.138349 | -2.75384 | -1.73587 | -2.38686 | -1.47714 | -2.96253 |
| 3-Phenyllactic acid | -0.31869 | 0.886174 | 0.586033 | 0.566315 | -0.51417 | 0.493947 | 0.537607 |
| 6-Methylnicotinamide | 1.221224 | -0.74783 | -0.8999 | -0.32875 | 0.750594 | -0.36423 | 0.209176 |
| 7-Ketocholesterol | -1.62193 | -0.72924 | -1.41262 | -0.67346 | -3.33838 | -1.05425 | 0.394191 |
| Adenosine | 1.160031 | -1.10972 | 3.551995 | 1.706158 | 2.330099 | 1.6743 | 1.64311 |
| Adrenic acid | -0.18514 | 0.31384 | 3.924449 | 1.628217 | 2.025823 | 1.822177 | 0.381029 |
| Aniline | 2.06256 | 0.099444 | 0.354683 | 1.012126 | 0.92077 | 0.789412 | 2.085041 |
| Ascorbic acid | 1.414815 | 0.241561 | -0.01993 | 0.763997 | 0.103 | 0.515553 | 1.813632 |
| Bilirubin | -0.03258 | 0.762612 | -2.80314 | -0.52063 | -2.86329 | -0.92415 | 1.033338 |
| Biotin | 0.354737 | 2.8532 | 2.548177 | 1.752961 | 1.674561 | 1.984084 | 0.478902 |
| Celestolide | -1.5588 | 4.012551 | 2.530276 | 1.552971 | 0.058632 | 1.838178 | -0.41259 |
| cis-2-Decenoic acid | -2.07923 | 0.770851 | 3.843393 | 2.00494 | -1.65831 | 1.746899 | 1.73883 |
| Citrulline | 0.747971 | -0.8549 | -2.32924 | -0.72048 | -1.32901 | -1.03297 | 0.792117 |
| dihydrotachysterol | -0.1759 | -1.50769 | 1.778932 | 0.424681 | 0.667147 | 0.394312 | 0.33649 |
| Docosapentaenoic acid | -1.27446 | 0.197547 | 2.508215 | 0.55958 | 0.906876 | 0.816236 | -0.92424 |
| Docosatrienoic acid | -1.00846 | -1.70877 | -1.38861 | -1.08454 | -1.84882 | -1.28764 | -0.36233 |
| Feruloylcholine | 1.854056 | 1.507357 | 3.227777 | 2.444553 | 2.333481 | 2.420855 | 2.330443 |
| gamma-Glutamylleucine | -0.94558 | 1.781421 | 0.149429 | -0.19815 | 0.451073 | 0.148611 | -1.70752 |
| gamma-Glutamyltyrosine | 3.077349 | -3.39858 | -2.34118 | -0.20786 | -0.82217 | -0.97008 | 3.461225 |
| Glutaconic acid | 1.708346 | 0.746083 | -2.26294 | 0.222426 | -1.43876 | -0.23636 | 2.264681 |
| Imidazoleacetic acid | 0.787501 | -0.29182 | -0.50125 | -0.00848 | 0.172243 | -0.10408 | 0.530099 |
| Jasmonic acid | -1.54304 | -0.4813 | -0.26023 | -1.08982 | -0.00975 | -0.80088 | -2.29994 |
| L-Ascorbate | 1.729686 | -0.29543 | 2.879405 | 2.253704 | 1.026388 | 1.900833 | 3.353833 |
| Lauric acid | -1.95862 | 0.233669 | 2.781121 | 0.441534 | 0.700494 | 0.76381 | -1.44161 |
| Lauric acid ethyl ester | -0.74582 | 6.622349 | 4.709755 | 3.043854 | 2.363482 | 3.643384 | -0.25483 |
| L-Dopa | -0.22379 | 0.156362 | -0.29498 | -0.15302 | -0.34775 | 0.423433 | 0.071336 |
| Lignoceric acid | 0.429934 | -0.78535 | 0.786058 | -0.41561 | 2.621649 | -0.00264 | -1.69723 |
| L-Kynurenine | -1.75347 | -1.69053 | 3.488484 | 0.027619 | 2.182862 | 0.508772 | -2.24453 |
| L-Serine | -2.33194 | 1.109858 | 0.647785 | 0.204644 | -2.25328 | 0.129347 | -0.28847 |
| Menaquinone | 3.034774 | 0.824367 | 0.464185 | 1.853179 | 0.662621 | 1.405184 | 3.7769 |
| Methyl palmitate | -0.45973 | 0.082958 | -1.88976 | -1.13315 | -0.68994 | -1.0565 | -1.25817 |
| Monoolein | -1.86161 | -2.64944 | 0.852193 | -0.23781 | -2.22612 | -0.58249 | 0.517687 |
| Muscone | 0.949917 | -0.78261 | 0.447791 | -0.03739 | 1.788533 | 0.109654 | -0.31731 |
| N,N-Dimethylarginine | 1.023607 | 3.310569 | -0.64162 | 0.844046 | 0.078122 | 0.852651 | 0.817369 |
| N1-Acetylspermine | 1.439215 | -2.36165 | -1.42963 | -0.47043 | -0.37897 | -0.83802 | 1.375476 |
| N8-Acetylspermidine | -1.50006 | -0.83649 | 0.646994 | -0.41174 | -0.49249 | -0.33061 | -1.0488 |
| N-Acetyl-DL-glutamic acid | 0.139513 | -3.44999 | -1.86169 | -1.03172 | -2.12174 | -1.56119 | 1.18958 |
| N-Acetylmannosamine | 2.053791 | 1.87147 | -2.72689 | 0.086503 | -0.67108 | -0.17564 | 1.550985 |
| N-Acetylneuraminic acid | -2.27168 | 1.214074 | 0.026238 | -1.00433 | 0.162989 | -0.48092 | -3.30075 |
| Oleic acid | 0.114873 | 0.772593 | 3.963952 | 1.229568 | 3.894117 | 1.812379 | -1.22481 |
| Orotic Acid | -0.488 | -0.24967 | -0.01845 | -0.19854 | -0.35295 | -0.20317 | -0.2996 |
| Ouabain | 0.085987 | 3.48579 | -0.14595 | 0.463394 | 0.671438 | 0.759473 | -0.7623 |
| Palmitoleic acid | 1.0199 | -0.80767 | -0.68526 | 0.406593 | -1.23272 | -0.06596 | 2.257324 |
| P-Aminobenzoate | 2.065743 | -0.38664 | 0.773845 | 0.643903 | 2.34689 | 0.693638 | 0.840646 |
| Phenylpyruvic acid | -1.37228 | -0.12709 | -0.7222 | -0.36779 | -2.29669 | -0.58579 | 0.095092 |
| Proline-hydroxyproline | -0.86422 | -1.67927 | 0.99713 | -0.08354 | -0.36532 | -0.1626 | -0.04915 |
| Propionyl-L-carnitine | 1.351705 | -1.42127 | -1.59838 | -0.81725 | 0.712855 | -0.86108 | -0.0714 |
| Punicic Acid | 2.190388 | -2.81008 | -5.12008 | -1.17419 | -3.78692 | -2.19768 | 3.452749 |
| Pyrrole-2-carboxylic acid | -1.29204 | 1.853184 | -2.57513 | -1.81019 | -0.16626 | -1.31712 | -3.49358 |
| S-Adenosylhomocysteine | 0.4678 | -1.37387 | -0.65336 | -0.94711 | 1.375763 | -0.73951 | -1.32475 |
| Sorbitan monostearate | 0.401184 | 3.862009 | 0.696381 | 0.939908 | 1.46332 | 1.306306 | -0.58512 |
| Spermidine | 2.018764 | 2.297326 | -1.57619 | 0.793187 | -0.47895 | 0.50914 | 2.125954 |
| Taurine | 0.733616 | 0.20211 | -0.76468 | -0.45126 | 1.154291 | -0.26725 | -0.81469 |
| Thiamine Pyrophosphate | 1.670637 | -2.63226 | -1.40256 | -0.86891 | 0.93698 | -0.99294 | 0.277813 |
| Thr-Leu | -3.75847 | -0.36066 | 2.905387 | -0.02901 | -0.80462 | 0.261133 | -2.1757 |
| Thymine | 0.640336 | 2.970952 | 1.348453 | 0.775216 | 2.831021 | 1.317966 | -1.26712 |
| trans-Aconitic acid | -0.6889 | -0.87843 | -1.40707 | -0.8943 | -1.43986 | -1.02704 | -0.392 |
| Uric acid | 0.075055 | -2.22489 | 0.826822 | 0.199007 | -0.36206 | -0.07306 | 1.121408 |
| Xanthine | 1.506213 | -2.6212 | -4.27252 | -3.13306 | 2.091703 | -2.72949 | -3.30031 |
| Xanthosine | -1.67646 | -3.84087 | -4.78734 | -3.15085 | -4.12468 | -3.57113 | -1.245 |

| Table S4. The coexistence probability of each differential metabolite and genus. | | | | | | | | | | | | | | | | | | | | | |
| --- | --- | --- | --- | --- | --- | --- | --- | --- | --- | --- | --- | --- | --- | --- | --- | --- | --- | --- | --- | --- | --- |
|  | *Actinomyces* | *Cutibacterium* | *Rubrobacter* | *Prevotella* | *Prevotella 7* | *Helicobacter* | *Bacillus* | *Gemella* | *Lysinibacillus* | *Streptococcus* | *Clostridium sensu stricto 1* | *Ezakiella* | *Parvimonas* | *Lachnoanaerobaculum* | *Ruminococcaceae UCG-014* | *Veillonella* | *Novosphingobium* | *Massilia* | *Serratia* | *Acinetobacter* | *Stenotrophomonas* |
| 10E,12Z-Octadecadienoic acid | -0.57663 | -0.25514 | -0.27861 | 0.470028 | -1.15764 | -0.26293 | -1.07268 | -1.34571 | -0.97783 | 2.294946 | 0.246307 | -1.07779 | -0.65845 | 2.859452 | -0.04472 | 1.071935 | 0.806936 | 0.464161 | -0.09703 | -0.36148 | 1.571873 |
| 1-Palmitoylglycerol | 0.696759 | 1.078291 | 0.120893 | 1.41047 | 0.701717 | 0.77361 | 0.513181 | -0.74027 | 0.312862 | 1.408066 | 1.359431 | 1.534086 | 0.232545 | 0.123663 | 0.446801 | 1.583458 | -0.08741 | 1.115947 | 1.291652 | 0.777388 | 0.961985 |
| 1-Stearoylglycerol | 2.986835 | 1.449907 | 0.262613 | 0.955352 | 2.371599 | 2.285113 | 2.456315 | 1.961711 | 0.726074 | -1.94628 | 0.714144 | 1.930335 | 2.101241 | -1.22911 | 0.842434 | 0.750599 | 0.476865 | 0.814027 | 1.363485 | 2.880314 | -0.96575 |
| 2-Aminoethanesulfinic Acid | 0.421535 | 0.648013 | -0.39597 | 0.964976 | 0.31646 | 0.440931 | 0.150329 | -1.12291 | -0.21274 | 0.793889 | 0.876482 | 1.07593 | -0.12689 | -0.32015 | -0.00696 | 1.155747 | -0.4942 | 0.667477 | 0.857824 | 0.503215 | 0.417042 |
| 2'-Deoxyinosine | -1.11706 | -0.91506 | 1.081399 | -0.9177 | -1.01298 | -0.75757 | -0.71435 | 1.359331 | 0.303224 | 0.926776 | -0.85421 | -2.13833 | 0.046666 | 2.817241 | 0.432002 | -0.93535 | 1.706906 | -0.40001 | -1.21024 | -1.11865 | 0.936352 |
| 2-Hydroxy-2-methylbutanedioic acid | 1.277765 | 0.471409 | 1.462291 | 0.7963 | 0.164726 | 1.326174 | 0.632697 | 2.03663 | 0.321571 | 2.197165 | 0.434682 | -1.26302 | 1.543601 | 5.404043 | 1.539453 | 1.29749 | 3.484194 | 1.200404 | 0.30694 | 1.417444 | 2.280448 |
| 3-Hydroxylidocaine | -3.10247 | -1.24407 | -0.37343 | -1.39646 | -1.32797 | -2.51987 | -1.70327 | -1.59083 | 0.200448 | -0.43173 | -0.81986 | -0.27898 | -2.05935 | -3.24402 | -1.21977 | -1.97661 | -2.47889 | -1.42775 | -1.27402 | -3.22816 | -0.9692 |
| 3-Ketodihydrosphingosine | 2.139763 | 1.022957 | 2.437796 | 0.888169 | 1.30301 | 2.032233 | 1.81875 | 3.892031 | 1.521 | 1.434721 | 0.635524 | -0.54084 | 2.67119 | 4.947623 | 2.277699 | 1.024135 | 4.062731 | 1.422725 | 0.674845 | 2.147181 | 2.040909 |
| 3-Phenyllactic acid | -0.77956 | -0.00119 | 0.563841 | -0.30822 | 0.272423 | -0.56843 | 0.093891 | 0.39746 | 1.004043 | -0.3638 | 0.030261 | 0.635796 | -0.15026 | -1.72917 | 0.047227 | -0.80244 | -0.74266 | -0.2819 | -0.10878 | -0.91175 | -0.36741 |
| 6-Methylnicotinamide | -0.42223 | -1.15851 | 0.59808 | -1.14454 | -1.13314 | -0.33685 | -0.62318 | 1.622158 | -0.48382 | 0.213864 | -1.35063 | -2.9101 | 0.356 | 3.644628 | 0.292672 | -0.93892 | 2.239674 | -0.5603 | -1.49278 | -0.38078 | 0.560343 |
| 7-Ketocholesterol | -2.5151 | -2.70364 | -2.04191 | -2.6576 | -2.73528 | -2.43661 | -2.55575 | -1.7525 | -2.45722 | -2.05697 | -2.7341 | -3.35172 | -2.1867 | -0.87481 | -2.16159 | -2.58161 | -1.45707 | -2.44746 | -2.82947 | -2.49285 | -1.98576 |
| Adenosine | -1.72265 | 0.661017 | 1.189757 | 0.163861 | 0.946924 | -1.15551 | 0.289897 | -0.15972 | 2.53327 | 0.122049 | 1.027238 | 2.753648 | -0.63133 | -4.66406 | 0.148078 | -0.88524 | -2.38666 | -0.05609 | 0.649953 | -1.98317 | -0.3367 |
| Adrenic acid | 6.038323 | 3.61507 | -1.24957 | 3.743998 | 4.09005 | 4.757328 | 3.91256 | -0.24153 | -0.27984 | -1.30202 | 2.965754 | 5.084502 | 2.77333 | -2.07476 | 0.947412 | 4.233028 | -0.36043 | 2.759662 | 4.114556 | 6.146902 | -0.63864 |
| Aniline | -1.25752 | -0.29403 | 0.134305 | -0.41989 | -0.26204 | -0.96418 | -0.47075 | -0.42711 | 0.499026 | -0.09018 | -0.10667 | 0.299435 | -0.69968 | -1.66084 | -0.31561 | -0.78355 | -1.07504 | -0.44544 | -0.32844 | -1.34001 | -0.32285 |
| Ascorbic acid | 1.887416 | -0.00703 | -2.93153 | -0.47305 | 1.022752 | 0.848866 | 0.904089 | -1.3086 | -1.89263 | -5.03138 | -0.84006 | 1.381669 | -0.0357 | -5.58195 | -1.71991 | -0.62451 | -3.01675 | -1.03791 | 0.131929 | 1.794438 | -3.99893 |
| Bilirubin | -0.29514 | -1.0497 | -0.11128 | -1.8999 | 0.086689 | -0.59031 | 0.252299 | 1.747611 | 0.165854 | -3.46231 | -1.75765 | -0.95526 | 0.266009 | -2.39045 | -0.54054 | -2.56285 | -0.59064 | -1.59135 | -1.45165 | -0.53573 | -2.4059 |
| Biotin | -0.35422 | -0.10313 | 1.39643 | -0.26911 | 0.020034 | -0.10269 | 0.179465 | 1.716884 | 1.046052 | 0.722815 | -0.12806 | -0.70229 | 0.598604 | 1.673799 | 0.810899 | -0.47411 | 1.407765 | 0.095474 | -0.3578 | -0.41339 | 0.834473 |
| Celestolide | -1.14882 | -0.7679 | 0.333734 | -1.28249 | -0.21203 | -1.02494 | -0.23993 | 0.858117 | 0.618023 | -1.48719 | -0.96337 | -0.47727 | -0.28318 | -1.82728 | -0.32029 | -1.87148 | -0.69991 | -1.06686 | -1.02891 | -1.3279 | -1.14407 |
| cis-2-Decenoic acid | -2.12247 | -0.05912 | 1.517289 | 0.114093 | -0.61063 | -1.28721 | -0.839 | -0.10552 | 1.487972 | 2.612489 | 0.617796 | 0.07585 | -0.73063 | 0.768513 | 0.543005 | -0.1712 | 0.158045 | 0.26776 | -0.10422 | -2.14773 | 1.745595 |
| Citrulline | -1.5929 | -1.87718 | -0.49174 | -1.98209 | -1.74846 | -1.47912 | -1.45218 | 0.200238 | -1.08565 | -1.11767 | -2.01618 | -2.87068 | -0.8614 | 0.825119 | -0.8655 | -2.00842 | 0.215661 | -1.57005 | -2.14648 | -1.61079 | -0.85667 |
| dihydrotachysterol | -1.90434 | -0.96236 | -0.82822 | -0.5908 | -1.52428 | -1.5114 | -1.6607 | -1.99819 | -0.92564 | 0.699874 | -0.4934 | -0.92307 | -1.6415 | -0.33155 | -0.96946 | -0.47206 | -1.12004 | -0.66253 | -0.85092 | -1.8278 | 0.044033 |
| Docosapentaenoic acid | 4.503618 | 1.611931 | -0.19532 | 1.872058 | 1.826256 | 3.583678 | 2.341747 | 1.692037 | -0.90528 | -0.16239 | 0.934666 | 0.427931 | 2.7218 | 3.692963 | 1.278366 | 2.696251 | 2.867749 | 1.797967 | 1.663212 | 4.692489 | 0.6764 |
| Docosatrienoic acid | -1.08637 | -0.52699 | -0.19096 | -0.21176 | -0.97702 | -0.78045 | -0.98962 | -0.92368 | -0.46906 | 1.001342 | -0.19546 | -0.81695 | -0.80298 | 0.83641 | -0.30862 | -0.05402 | -0.06495 | -0.17832 | -0.48777 | -1.01076 | 0.546793 |
| Feruloylcholine | 0.391853 | -0.6615 | 2.340712 | -1.22062 | 0.047063 | 0.405156 | 0.723962 | 4.547088 | 1.254535 | -0.23931 | -1.26539 | -2.64826 | 1.905494 | 4.26394 | 1.505744 | -1.46303 | 3.72527 | -0.28733 | -1.32161 | 0.269838 | 0.7574 |
| gamma-Glutamylleucine | -1.33471 | 0.235548 | 1.371637 | 0.181344 | 0.056605 | -0.75936 | -0.17208 | 0.346261 | 1.595663 | 1.54365 | 0.639191 | 0.663857 | -0.2522 | -0.24924 | 0.569933 | -0.22805 | -0.07936 | 0.26914 | 0.164796 | -1.41519 | 1.042709 |
| gamma-Glutamyltyrosine | -1.19575 | -1.42494 | -1.10502 | -1.1238 | -1.74677 | -1.10161 | -1.55584 | -1.14089 | -1.68642 | -0.21711 | -1.32082 | -2.24581 | -1.12924 | 1.078544 | -1.00105 | -0.79984 | -0.10023 | -0.98544 | -1.45107 | -1.09187 | -0.35827 |
| Glutaconic acid | 1.658199 | -0.10159 | -0.67475 | -0.18315 | 0.309431 | 1.110464 | 0.659514 | 0.871727 | -1.05399 | -1.55717 | -0.6533 | -0.82372 | 0.934058 | 0.985229 | -0.03685 | 0.087475 | 0.921134 | -0.0993 | -0.2066 | 1.697485 | -0.8194 |
| Imidazoleacetic acid | 3.209354 | 0.2639 | -2.07601 | 0.223714 | 0.884286 | 2.108767 | 1.249085 | 0.092027 | -2.22512 | -3.07156 | -0.60788 | -0.14001 | 1.205855 | -0.23108 | -0.55785 | 0.74884 | 0.194512 | -0.0075 | 0.323315 | 3.300682 | -1.9766 |
| Jasmonic acid | -1.3192 | 1.599277 | 0.888301 | 1.926727 | 0.861498 | -0.54766 | 0.040458 | -2.11801 | 2.029767 | 2.965688 | 2.584458 | 3.702765 | -0.96787 | -2.87815 | 0.421478 | 1.522268 | -2.1636 | 1.344745 | 1.984421 | -1.34445 | 1.560592 |
| L-Ascorbate | 0.147543 | -0.16401 | 0.421603 | -0.07568 | -0.22651 | 0.195543 | -0.01758 | 0.753988 | -0.06097 | 0.519872 | -0.20463 | -0.91029 | 0.390818 | 1.9201 | 0.377676 | 0.067414 | 1.202335 | 0.13349 | -0.28007 | 0.188061 | 0.599721 |
| Lauric acid | 3.964507 | 1.818085 | -2.88501 | 1.882321 | 2.325462 | 2.759787 | 2.087357 | -2.01043 | -1.79533 | -3.08522 | 1.209305 | 3.469682 | 0.877806 | -4.3086 | -0.84243 | 2.249885 | -2.37828 | 0.899421 | 2.30343 | 4.041719 | -2.46745 |
| Lauric acid ethyl ester | 4.615817 | 3.421314 | -2.23196 | 3.526653 | 3.796395 | 3.533112 | 3.147952 | -2.4235 | -0.38182 | -1.9105 | 3.092951 | 6.351067 | 1.263314 | -5.97119 | -0.15717 | 3.685615 | -3.16794 | 2.153321 | 4.129447 | 4.660757 | -1.72539 |
| L-Dopa | 0.608049 | 0.091129 | -0.36538 | -1.07161 | -0.64648 | -1.11254 | -1.00954 | -1.5619 | 0.650792 | -0.15187 | -0.04191 | 0.799426 | -1.81008 | -0.63884 | -0.88971 | 0.12223 | -0.83754 | -1.18698 | 1.545272 | 0.545756 | 0.038252 |
| Lignoceric acid | -2.11334 | -0.75021 | 1.102633 | -1.41767 | -0.18339 | -1.67019 | -0.37684 | 1.102311 | 1.64533 | -0.98759 | -0.75566 | -0.05136 | -0.53959 | -2.593 | -0.11923 | -2.3568 | -1.05091 | -1.14342 | -1.07062 | -2.37798 | -0.86492 |
| L-Kynurenine | -0.11025 | 0.46983 | 0.166891 | 0.702013 | 0.15356 | 0.083581 | 0.003445 | -0.61919 | 0.272852 | 1.121863 | 0.753313 | 0.76237 | -0.14292 | 0.043297 | 0.198193 | 0.752923 | -0.1863 | 0.552509 | 0.584589 | -0.06608 | 0.707315 |
| L-Serine | 0.987009 | 0.769766 | 1.197484 | 0.047302 | 1.701339 | 0.795519 | 1.664687 | 2.3406 | 1.77052 | -1.49563 | 0.281268 | 1.411711 | 1.384363 | -1.82007 | 0.823892 | -0.63021 | 0.130265 | 0.149057 | 0.512068 | 0.761961 | -0.77103 |
| Menaquinone | -4.273 | -1.86213 | 0.510542 | -2.13946 | -1.94994 | -3.36564 | -2.23794 | -0.73776 | 0.804904 | 0.086701 | -1.34469 | -1.35882 | -2.241 | -2.2618 | -0.99415 | -2.91955 | -1.82461 | -1.84486 | -2.08827 | -4.45278 | -0.50399 |
| Methyl palmitate | -0.1872 | -0.8702 | -2.74668 | 0.410063 | -2.21676 | -0.23895 | -2.05829 | -3.95283 | -3.70358 | 1.574124 | -0.33968 | -1.95279 | -1.6814 | 2.856147 | -1.43617 | 1.725186 | -0.17807 | 0.072541 | -0.41149 | 0.237074 | 0.700943 |
| Monoolein | -1.03539 | -0.43243 | -0.96389 | -0.0114 | -0.97222 | -0.81263 | -1.12368 | -2.03173 | -0.9696 | 0.674967 | -0.0344 | -0.24698 | -1.23452 | -0.39236 | -0.78931 | 0.211737 | -1.05676 | -0.21474 | -0.24303 | -0.93285 | 0.083782 |
| Muscone | -2.02745 | -2.18193 | 0.242615 | -2.45483 | -1.92024 | -1.77895 | -1.51584 | 1.225186 | -0.55392 | -1.00327 | -2.37409 | -3.55327 | -0.65819 | 1.604203 | -0.55046 | -2.64157 | 0.916134 | -1.78721 | -2.62494 | -2.09845 | -0.61337 |
| N,N-Dimethylarginine | -1.70374 | -1.29801 | -2.48922 | -0.69746 | -1.99597 | -1.57473 | -2.16822 | -3.72506 | -2.49903 | -0.23942 | -0.85562 | -1.06349 | -2.35785 | -1.38745 | -1.98449 | -0.27484 | -2.27745 | -1.04244 | -0.99227 | -1.53926 | -0.93016 |
| N1-Acetylspermine | 0.228804 | 0.319525 | 1.246869 | 0.168198 | 0.477765 | 0.359864 | 0.582786 | 1.573206 | 1.062236 | 0.613678 | 0.253788 | -0.01873 | 0.827049 | 1.237117 | 0.883759 | -0.00435 | 1.219806 | 0.399559 | 0.136712 | 0.177563 | 0.754312 |
| N8-Acetylspermidine | 1.381073 | 0.133514 | 1.057759 | 0.296378 | 0.091526 | 1.256786 | 0.626602 | 2.186047 | -0.04737 | 1.08782 | -0.12081 | -1.62767 | 1.535196 | 4.786447 | 1.209559 | 0.737765 | 3.228538 | 0.733716 | -0.09197 | 1.486663 | 1.474918 |
| N-Acetyl-DL-glutamic acid | -0.63818 | -0.59806 | -0.67789 | -0.41869 | -0.78597 | -0.5682 | -0.76584 | -0.88992 | -0.83118 | -0.0991 | -0.49349 | -0.76713 | -0.68212 | 0.019013 | -0.58334 | -0.28861 | -0.41399 | -0.43501 | -0.57072 | -0.58711 | -0.25432 |
| N-Acetylmannosamine | -1.30943 | -1.3905 | -0.86289 | -1.49269 | -1.24733 | -1.26652 | -1.15518 | -0.52497 | -1.00197 | -1.35134 | -1.47866 | -1.64727 | -0.9852 | -0.77743 | -1.03694 | -1.5908 | -0.76742 | -1.3436 | -1.51976 | -1.34013 | -1.2005 |
| N-Acetylneuraminic acid | 0.928725 | 0.454631 | -0.04839 | 0.402417 | 0.646445 | 0.734544 | 0.673907 | 0.363591 | 0.040317 | -0.41546 | 0.277775 | 0.558135 | 0.580098 | -0.1933 | 0.217803 | 0.409703 | 0.147328 | 0.327606 | 0.451253 | 0.924228 | -0.1925 |
| Oleic acid | -0.70918 | -0.3234 | -0.91751 | 0.091027 | -0.82141 | -0.55401 | -0.92769 | -1.80218 | -0.97821 | 0.632224 | 0.009928 | -0.23969 | -0.99862 | -0.10434 | -0.6657 | 0.355391 | -0.79985 | -0.09952 | -0.14424 | -0.60008 | 0.127611 |
| Orotic Acid | -0.47152 | 0.484071 | 2.710031 | 0.375144 | 0.399662 | 0.08583 | 0.512938 | 2.502211 | 2.2684 | 2.41229 | 0.694212 | -0.20159 | 1.046127 | 2.951691 | 1.73322 | 0.088343 | 2.344588 | 0.843599 | 0.195304 | -0.53733 | 2.231267 |
| Ouabain | 1.349627 | 1.380843 | 0.795439 | 1.557198 | 1.225068 | 1.342977 | 1.158013 | 0.491629 | 0.862283 | 1.397327 | 1.476936 | 1.560954 | 1.032299 | 0.946144 | 1.042078 | 1.669915 | 0.838506 | 1.403608 | 1.479178 | 1.397033 | 1.230362 |
| Palmitoleic acid | -2.5319 | -1.4 | -0.78676 | -1.21081 | -1.78952 | -2.07419 | -1.93562 | -1.81593 | -0.80107 | 0.131124 | -0.9806 | -1.2853 | -1.9124 | -1.01814 | -1.20342 | -1.29854 | -1.461 | -1.18948 | -1.38024 | -2.51901 | -0.42629 |
| P-Aminobenzoate | -1.81569 | -0.99647 | 1.046572 | -1.0427 | -1.12925 | -1.30176 | -0.99695 | 0.848293 | 0.554854 | 0.936817 | -0.78919 | -1.74615 | -0.44955 | 1.631233 | 0.189967 | -1.24285 | 0.886947 | -0.60129 | -1.25694 | -1.85677 | 0.748541 |
| Phenylpyruvic acid | 3.64867 | 1.63284 | -0.26431 | 0.883093 | 2.973824 | 2.643332 | 3.0005 | 1.996703 | 0.608282 | -3.46938 | 0.609287 | 2.645387 | 2.319983 | -3.08473 | 0.552518 | 0.515024 | -0.33536 | 0.59842 | 1.55462 | 3.475774 | -2.11426 |
| Proline-hydroxyproline | -0.68237 | 0.57038 | 1.775979 | 0.970628 | -0.12782 | -0.04442 | -0.1518 | 0.521895 | 1.306935 | 3.427927 | 1.151386 | 0.044773 | 0.232449 | 3.084245 | 1.239534 | 1.08116 | 1.659384 | 1.150427 | 0.56003 | -0.60307 | 2.6663 |
| Propionyl-L-carnitine | -1.24146 | -0.38299 | 1.408033 | -0.78938 | -0.06405 | -0.861 | -0.07209 | 1.535148 | 1.463377 | 0.070602 | -0.37692 | -0.39161 | 0.094637 | -0.20514 | 0.460013 | -1.35936 | 0.349674 | -0.44508 | -0.68056 | -1.40149 | 0.163372 |
| Punicic Acid | 3.143206 | 1.706881 | -1.10071 | 1.449004 | 2.410111 | 2.302767 | 2.239638 | -0.08877 | -0.16619 | -2.33275 | 1.123458 | 3.030643 | 1.351227 | -3.272 | 0.036394 | 1.400254 | -1.2372 | 0.865195 | 1.91083 | 3.09874 | -1.64787 |
| Pyrrole-2-carboxylic acid | -0.53523 | -1.29989 | -0.58113 | -0.99194 | -1.5769 | -0.52052 | -1.17186 | -0.07906 | -1.56621 | 0.127768 | -1.3372 | -2.79029 | -0.40002 | 2.913875 | -0.44187 | -0.52573 | 1.236457 | -0.66918 | -1.42558 | -0.40439 | 0.199888 |
| S-Adenosylhomocysteine | 0.04592 | -0.29162 | 0.666002 | -0.01791 | -0.59884 | 0.196303 | -0.27356 | 0.885259 | -0.20611 | 1.376783 | -0.22824 | -1.59169 | 0.43336 | 3.565912 | 0.598461 | 0.324747 | 2.023842 | 0.308345 | -0.42374 | 0.149381 | 1.322676 |
| Sorbitan monostearate | 2.597305 | 2.200826 | 0.260905 | 2.733305 | 1.738839 | 2.388547 | 1.63219 | -0.35388 | 0.32445 | 2.06617 | 2.374384 | 2.484008 | 1.343021 | 1.47177 | 1.186438 | 3.248579 | 0.930229 | 2.291308 | 2.533499 | 2.768825 | 1.714086 |
| Spermidine | 1.838696 | 0.606617 | 1.353651 | 0.775339 | 0.564782 | 1.694447 | 1.065995 | 2.425743 | 0.325303 | 1.422017 | 0.358524 | -1.02922 | 1.900168 | 4.897271 | 1.556487 | 1.213154 | 3.439666 | 1.162397 | 0.409013 | 1.944447 | 1.792021 |
| Taurine | 0.365398 | 0.446412 | 0.053342 | 0.210724 | 0.780908 | 0.277106 | 0.636444 | 0.215428 | 0.538644 | -0.78243 | 0.317701 | 1.144084 | 0.273411 | -1.80029 | 0.050577 | -0.08515 | -0.72359 | 0.076861 | 0.433905 | 0.279421 | -0.6129 |
| Thiamine Pyrophosphate | -1.17093 | -0.90865 | 1.119678 | -0.89526 | -1.03642 | -0.78636 | -0.74441 | 1.333534 | 0.333876 | 1.038331 | -0.82144 | -2.13492 | 0.02191 | 2.877481 | 0.45065 | -0.90999 | 1.722382 | -0.37627 | -1.20004 | -1.16969 | 1.012373 |
| Thr-Leu | -0.12326 | -0.811 | -0.52806 | -0.22996 | -1.4117 | -0.07061 | -1.03876 | -0.49291 | -1.61056 | 1.191671 | -0.67137 | -2.35183 | -0.27362 | 3.789402 | -0.16256 | 0.471855 | 1.568255 | -0.02001 | -0.81008 | 0.089523 | 0.987879 |
| Thymine | -0.74462 | -0.07955 | 0.150198 | 0.068691 | -0.33291 | -0.47628 | -0.43199 | -0.5054 | 0.157391 | 0.800959 | 0.183073 | 0.029218 | -0.44596 | 0.031633 | -0.03774 | 0.035277 | -0.23729 | 0.047768 | -0.05187 | -0.72775 | 0.443579 |
| trans-Aconitic acid | -3.47861 | -1.74723 | -0.94524 | -1.54837 | -2.24241 | -2.83458 | -2.51248 | -2.46703 | -0.80468 | 0.187665 | -1.15408 | -1.34995 | -2.59907 | -1.87078 | -1.5835 | -1.75773 | -2.21112 | -1.55857 | -1.70327 | -3.48647 | -0.60092 |
| Uric acid | 1.457193 | 2.22299 | -0.49947 | 2.431564 | 2.039977 | 1.340999 | 1.409724 | -1.91243 | 0.76532 | 0.608149 | 2.523979 | 4.363433 | 0.17993 | -3.64552 | 0.191156 | 2.338133 | -2.19336 | 1.592624 | 2.694288 | 1.471331 | 0.057718 |
| Xanthine | 0.342627 | 2.145679 | -0.14 | 1.907358 | 2.345025 | 0.426941 | 1.409423 | -1.85413 | 1.886687 | -0.28856 | 2.477897 | 5.391198 | -0.30331 | -6.76834 | -0.06591 | 1.153082 | -3.76254 | 1.014818 | 2.544737 | 0.172462 | -0.84263 |
| Xanthosine | -2.11169 | -1.6313 | -1.0065 | -2.01253 | -1.22793 | -1.99151 | -1.33436 | -0.81759 | -0.62908 | -2.29463 | -1.72538 | -1.13774 | -1.51079 | -3.14822 | -1.48876 | -2.50925 | -2.09336 | -1.93288 | -1.78761 | -2.25495 | -2.1192 |
