# Supplementary figures and images for: Microbial-Related Metabolites May Be Involved in Eight Major Biological Processes and Represent Potential Diagnostic Markers in Gastric Cancer
Source: Cancers (Basel). 2023 Nov 3;15(21):5271. doi: 10.3390/cancers15215271 (PMC10649575; doi:10.3390/cancers15215271)

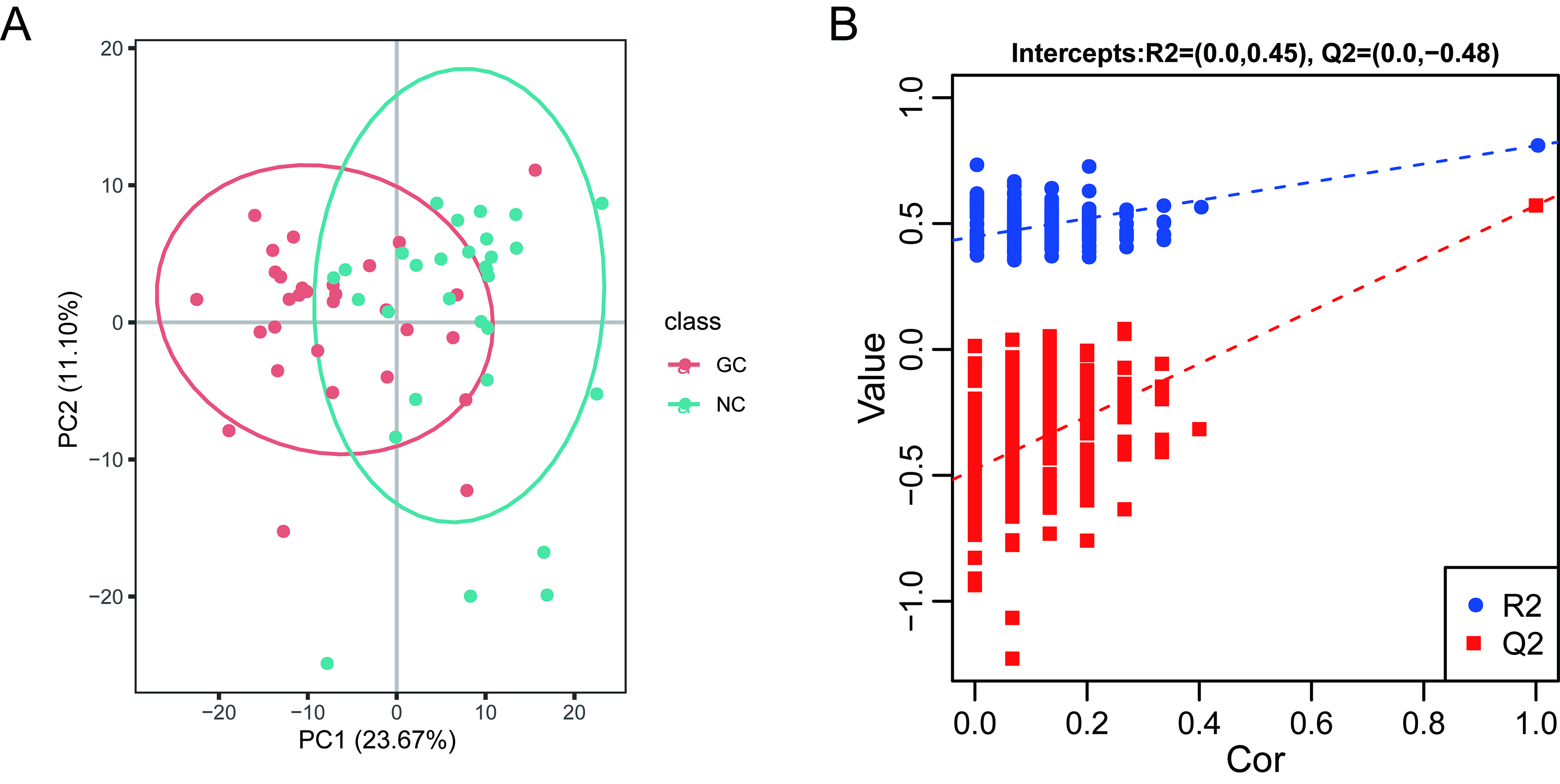

Supplement: Supplementary file 1 [file cancers-15-05271-s001.zip › Supplemental Material - cancers/Figure S1.tif]

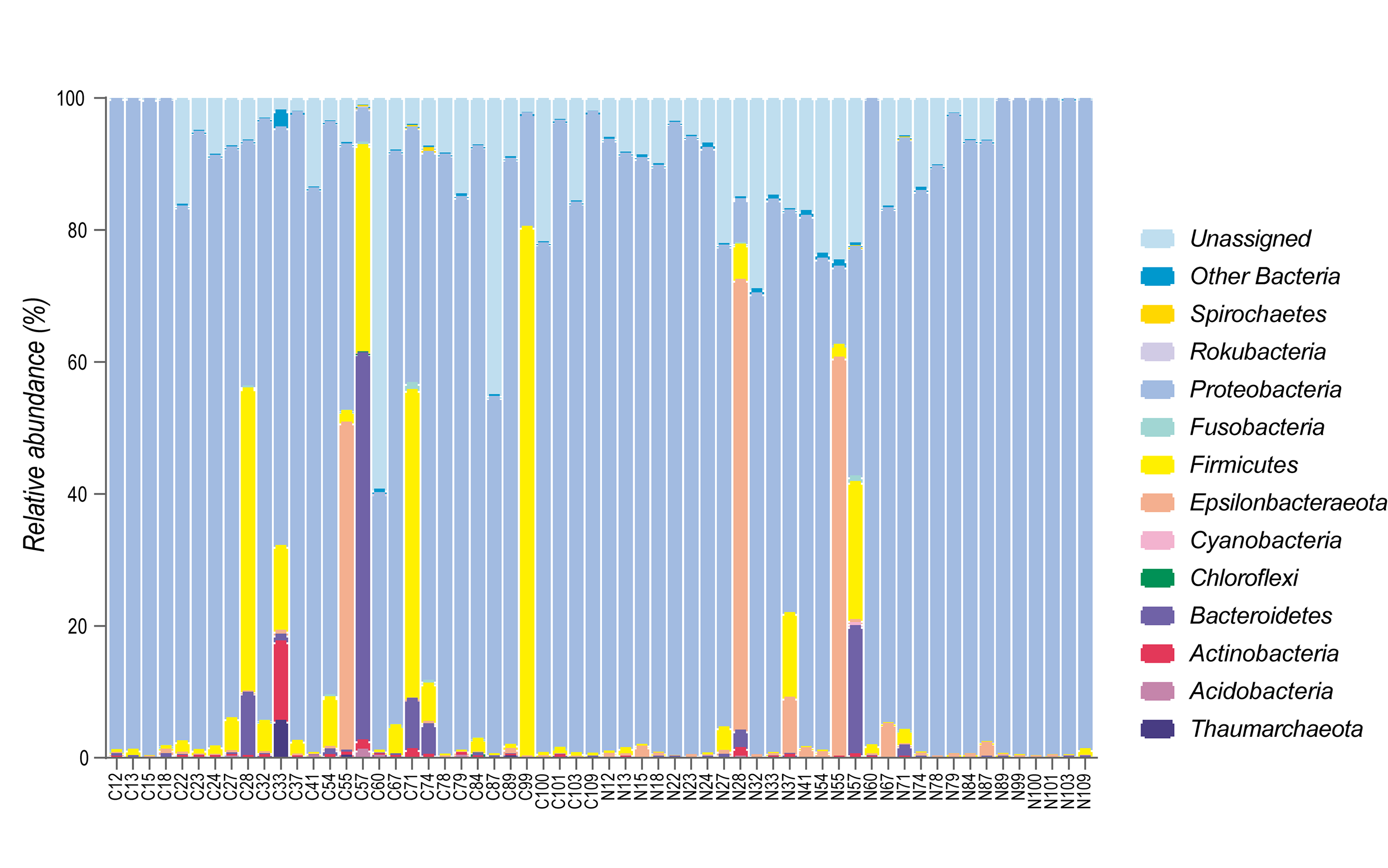

Supplement: Supplementary file 1 [file cancers-15-05271-s001.zip › Supplemental Material - cancers/Figure S2.tif]

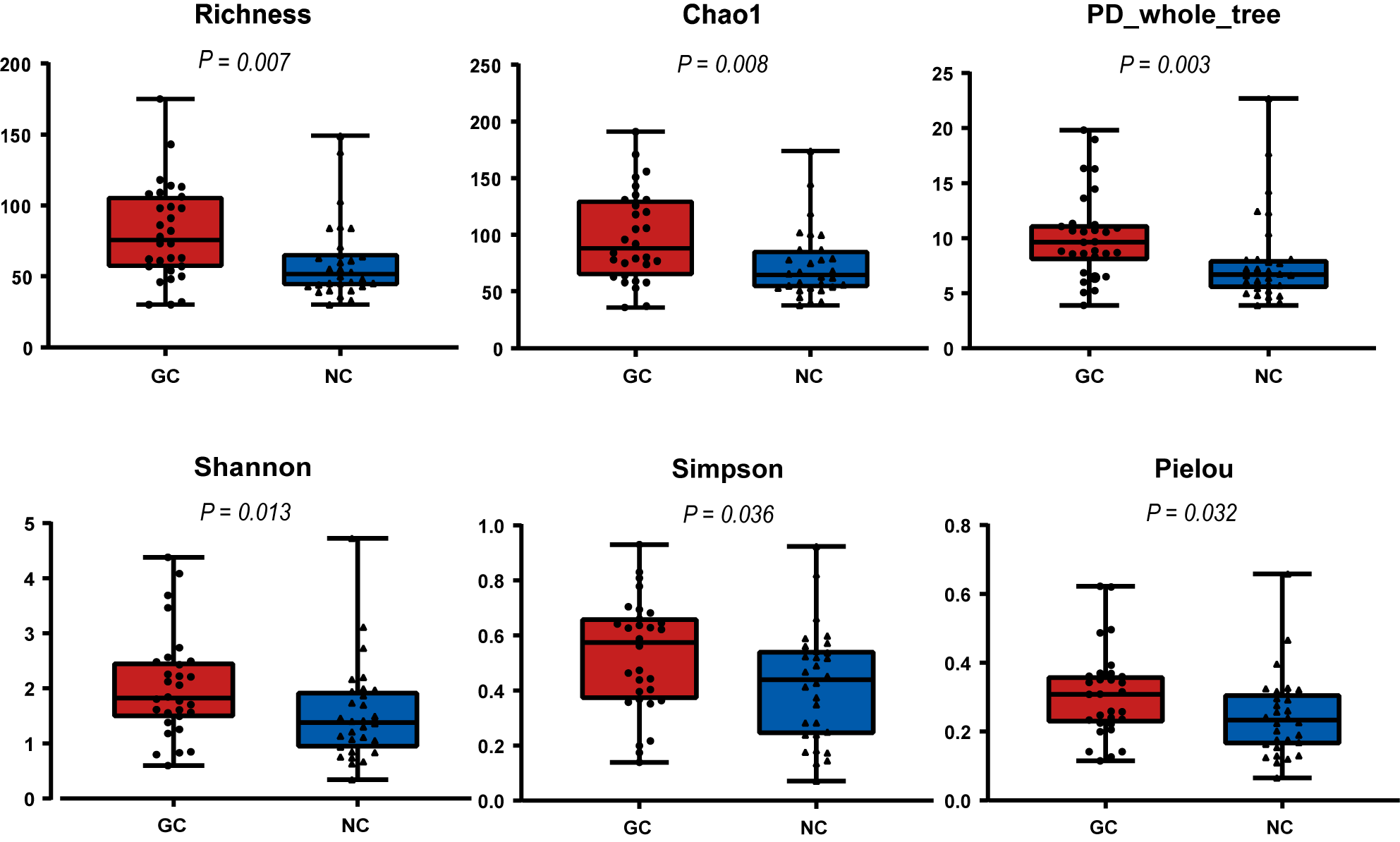

Supplement: Supplementary file 1 [file cancers-15-05271-s001.zip › Supplemental Material - cancers/Figure S3.tif]

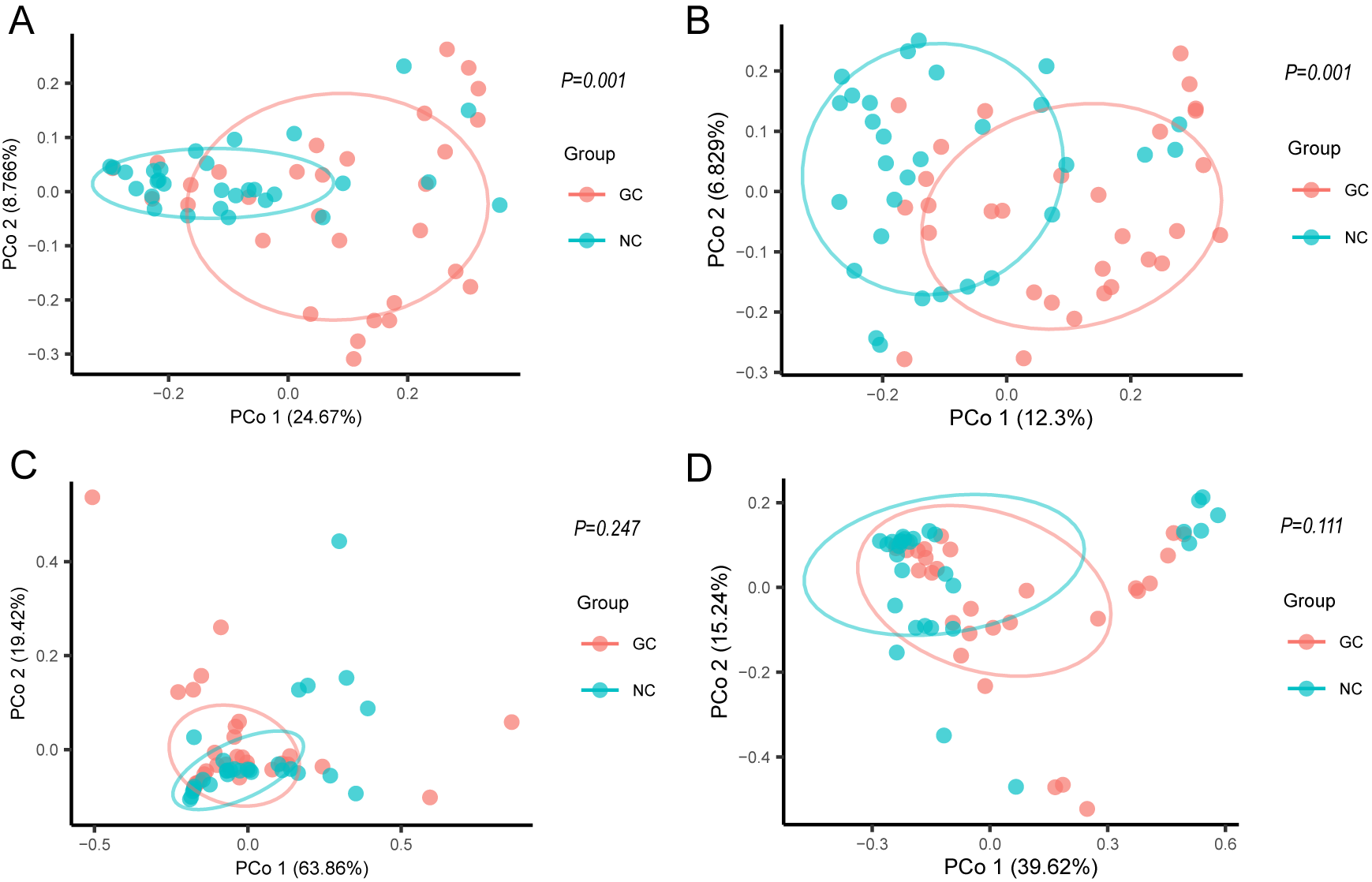

Supplement: Supplementary file 1 [file cancers-15-05271-s001.zip › Supplemental Material - cancers/Figure S4.tif]

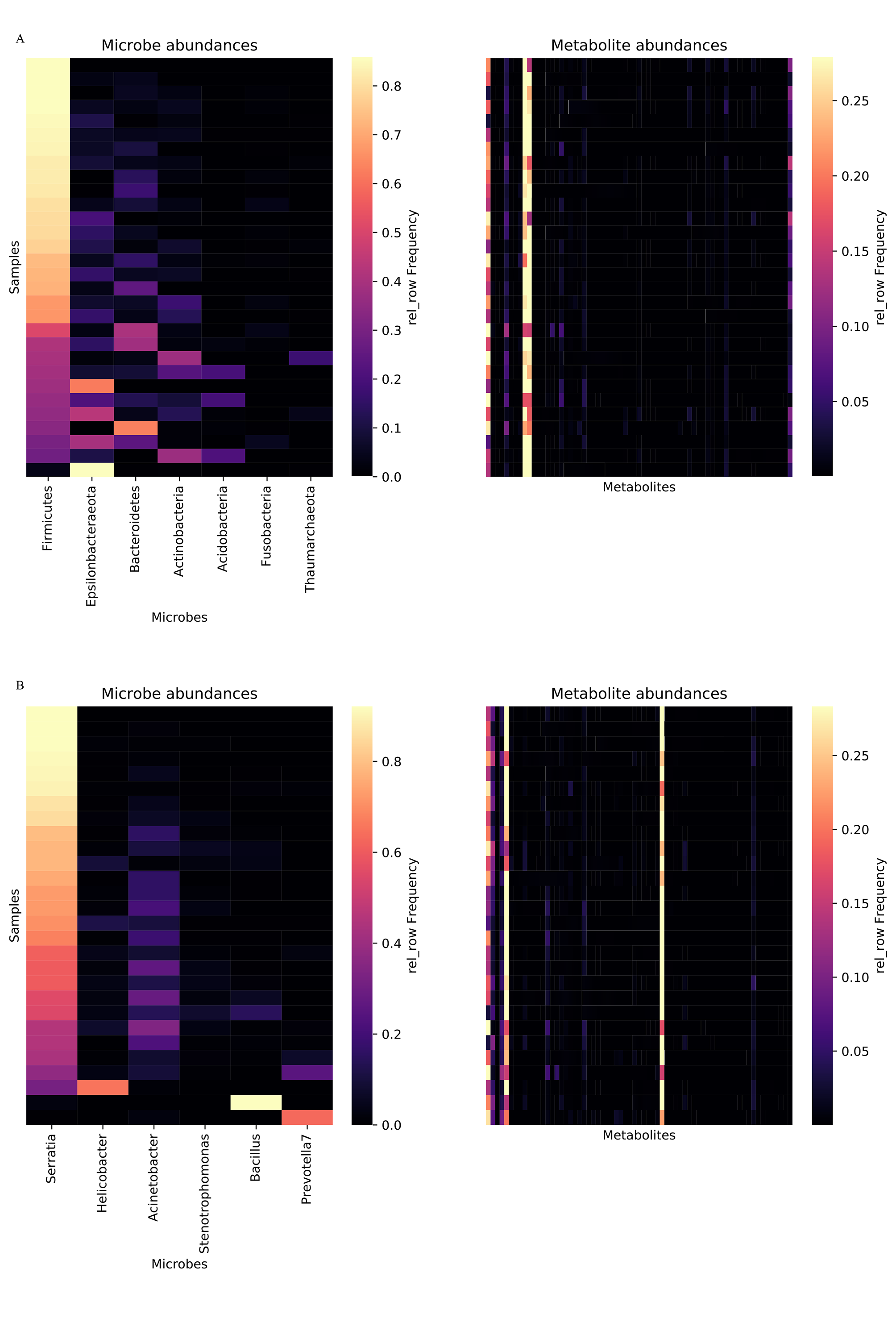

Supplement: Supplementary file 1 [file cancers-15-05271-s001.zip › Supplemental Material - cancers/Figure S5.tif]

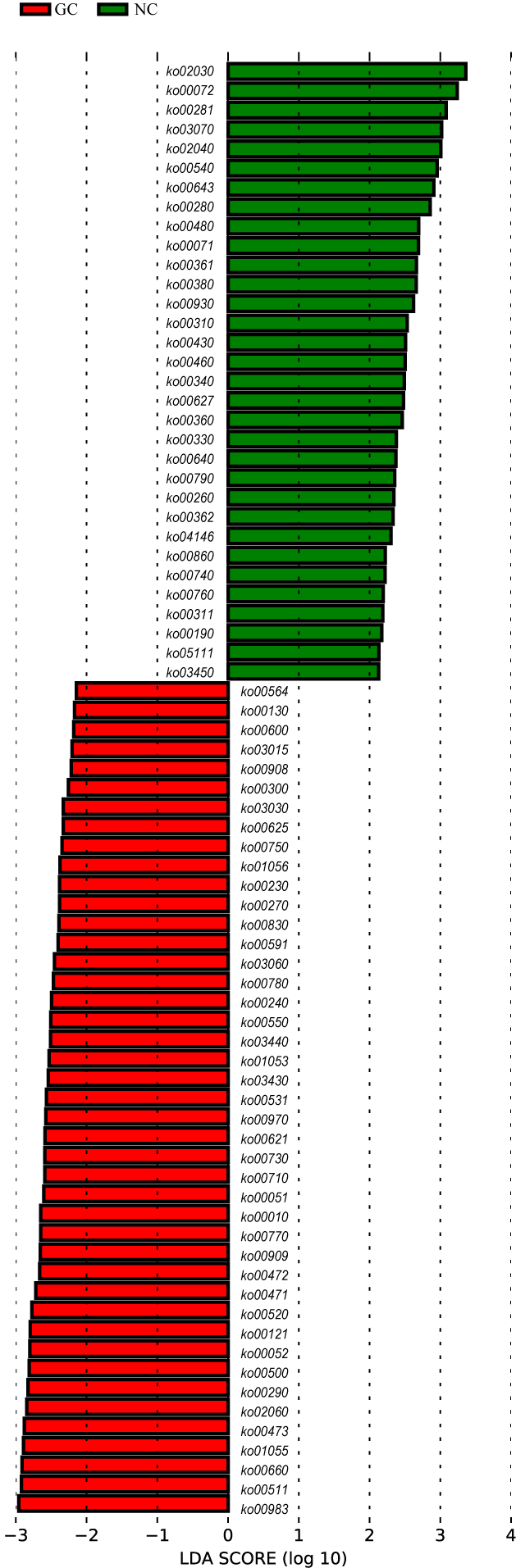

Supplement: Supplementary file 1 [file cancers-15-05271-s001.zip › Supplemental Material - cancers/Figure S6.tif]
